# Correction: Immunosuppressive FK506 treatment leads to more frequent EBV-associated lymphoproliferative disease in humanized mice

**DOI:** 10.1371/journal.ppat.1009167

**Published:** 2020-12-21

**Authors:** Nicole Caduff, Donal McHugh, Anita Murer, Patrick Rämer, Ana Raykova, Vanessa Landtwing, Lisa Rieble, Christian W. Keller, Michael Prummer, Laurent Hoffmann, Janice K. P. Lam, Alan K. S. Chiang, Friedrich Raulf, Tarik Azzi, Christoph Berger, Tina Rubic-Schneider, Elisabetta Traggiai, Jan D. Lünemann, Michael Kammüller, Christian Münz

The following information is missing from the Funding statement: AKSC was supported by a HMRF grant (18170462).

## References

[1] Caduff N, McHugh D, Murer A, Rämer P, Raykova A, Landtwing V, et al (2020) Immunosuppressive FK506 treatment leads to more frequent EBV-associated lymphoproliferative disease in humanized mice. PLoS Pathog 16(4): e1008477 10.1371/journal.ppat.1008477 32251475 PMC7162544

