# Supplementary material for: Immunosuppressive FK506 treatment leads to more frequent EBV-associated lymphoproliferative disease in humanized mice
Source: PLoS Pathog. 2020 Apr 6;16(4):e1008477. doi: 10.1371/journal.ppat.1008477 (PMC7162544; doi:10.1371/journal.ppat.1008477)
Supplement: S1 Table — (PDF) [file ppat.1008477.s002.pdf]

S1 Table. Curated gene set list for GSEA.

Collection	Molecular Signatures Database Gene Set Name
C2	ABRAHAM_ALPC_VS_MULTIPLE_MYELOMA_DN
C2	ABRAHAM_ALPC_VS_MULTIPLE_MYELOMA_UP
C2	ACOSTA_PROLIFERATION_INDEPENDENT_MYC_TARGETS_DN
C2	ACOSTA_PROLIFERATION_INDEPENDENT_MYC_TARGETS_UP
C2	ALCALA_APOPTOSIS
C2	BASSO_B_LYMPHOCYTE_NETWORK
C2	BASSO_CD40_SIGNALING_DN
C2	BASSO_CD40_SIGNALING_UP
C2	BASSO_HAIRY_CELL_LEUKEMIA_DN
C2	BASSO_HAIRY_CELL_LEUKEMIA_UP
C2	BILBAN_B_CLL_LPL_DN
C2	BILBAN_B_CLL_LPL_UP
C2	BILD_MYC_ONCOGENIC_SIGNATURE
C2	BIOCARTA_ASBCELL_PATHWAY
C2	BIOCARTA_BCELLSURVIVAL_PATHWAY
C2	BIOCARTA_BCR_PATHWAY
C2	BIOCARTA_BLYMPHOCYTE_PATHWAY
C2	BIOCARTA_CASPASE_PATHWAY
C2	BIOCARTA_CELLCYCLE_PATHWAY
C2	BIOCARTA_CHEMICAL_PATHWAY
C2	BIOCARTA_DEATH_PATHWAY
C2	BIOCARTA_DNAFRAGMENT_PATHWAY
C2	BIOCARTA_FREE_PATHWAY
C2	BIOCARTA_G1_PATHWAY
C2	BIOCARTA_G2_PATHWAY
C2	BIOCARTA_IGF1R_PATHWAY
C2	BIOCARTA_MITOCHONDRIA_PATHWAY
C2	BIOCARTA_P27_PATHWAY
C2	BIOCARTA_PTC1_PATHWAY
C2	BIOCARTA_PTEN_PATHWAY
C2	BIOCARTA_RANMS_PATHWAY
C2	BIOCARTA_SET_PATHWAY
C2	BIOCARTA_TALL1_PATHWAY
C2	BOHN_PRIMARY_IMMUNODEFICIENCY_SYNDROM_DN

C2	BOHN_PRIMARY_IMMUNODEFICIENCY_SYNDROM_UP
C2	BROCKE_APOPTOSIS_REVERSED_BY_IL6
C2	CEBALLOS_TARGETS_OF_TP53_AND_MYC_DN
C2	CEBALLOS_TARGETS_OF_TP53_AND_MYC_UP
C2	DANG_BOUND_BY_MYC
C2	DANG_MYC_TARGETS_DN
C2	DANG_MYC_TARGETS_UP
C2	DANG_REGULATED_BY_MYC_DN
C2	DANG_REGULATED_BY_MYC_UP
C2	DIRMEIER_LMP1_RESPONSE_EARLY
C2	DIRMEIER_LMP1_RESPONSE_LATE_DN
C2	DIRMEIER_LMP1_RESPONSE_LATE_UP
C2	DORSEY_GAB2_TARGETS
C2	DUTTA_APOPTOSIS_VIA_NFKB
C2	EGUCHI_CELL_CYCLE_RB1_TARGETS
C2	EHRLICH_ICF_SYNDROM_DN
C2	EHRLICH_ICF_SYNDROM_UP
C2	FAELT_B_CLL_WITH_VH_REARRANGEMENTS_DN
C2	FAELT_B_CLL_WITH_VH_REARRANGEMENTS_UP
C2	FAELT_B_CLL_WITH_VH3_21_DN
C2	FAELT_B_CLL_WITH_VH3_21_UP
C2	FERNANDEZ_BOUND_BY_MYC
C2	FLECHNER_PBL_KIDNEY_TRANSPLANT_OK_VS_DONOR_DN
C2	FLECHNER_PBL_KIDNEY_TRANSPLANT_OK_VS_DONOR_UP
C2	FLECHNER_PBL_KIDNEY_TRANSPLANT_REJECTED_VS_OK_DN
C2	FLECHNER_PBL_KIDNEY_TRANSPLANT_REJECTED_VS_OK_UP
C2	GARY_CD5_TARGETS_DN
C2	GARY_CD5_TARGETS_UP
C2	GOTTWEIN_TARGETS_OF_KSHV_MIR_K12_11
C2	GUTIERREZ_CHRONIC_LYMPHOCYTIC_LEUKEMIA_DN
C2	GUTIERREZ_CHRONIC_LYMPHOCYTIC_LEUKEMIA_UP
C2	GUTIERREZ_MULTIPLE_MYELOMA_UP
C2	GUTIERREZ_WALDENSTROEMS_MACROGLOBULINEMIA_1_DN
C2	GUTIERREZ_WALDENSTROEMS_MACROGLOBULINEMIA_1_UP
C2	GUTIERREZ_WALDENSTROEMS_MACROGLOBULINEMIA_2
C2	HADDAD_B_LYMPHOCYTE_PROGENITOR

C2	HASLINGER_B_CLL_WITH_11Q23_DELETION
C2	HASLINGER_B_CLL_WITH_13Q14_DELETION
C2	HASLINGER_B_CLL_WITH_17P13_DELETION
C2	HASLINGER_B_CLL_WITH_6Q21_DELETION
C2	HASLINGER_B_CLL_WITH_CHROMOSOME_12_TRISOMY
C2	HASLINGER_B_CLL_WITH_MUTATED_VH_GENES
C2	HOEGERKORP_CD44_TARGETS_DIRECT_DN
C2	HOEGERKORP_CD44_TARGETS_DIRECT_UP
C2	HOEGERKORP_CD44_TARGETS_TEMPORAL_DN
C2	HOEGERKORP_CD44_TARGETS_TEMPORAL_UP
C2	HOFMANN_CELL_LYMPHOMA_DN
C2	HOFMANN_CELL_LYMPHOMA_UP
C2	HOLLMANN_APOPTOSIS_VIA_CD40_DN
C2	HOLLMANN_APOPTOSIS_VIA_CD40_UP
C2	HUMMEL_BURKITTS_LYMPHOMA_DN
C2	HUMMEL_BURKITTS_LYMPHOMA_UP
C2	HUTTMANN_B_CLL_POOR_SURVIVAL_DN
C2	HUTTMANN_B_CLL_POOR_SURVIVAL_UP
C2	JAIN_NFKB_SIGNALING
C2	KEGG_APOPTOSIS
C2	KEGG_B_CELL_RECEPTOR_SIGNALING_PATHWAY
C2	KEGG_CELL_CYCLE
C2	KLEIN_PRIMARY_EFFUSION_LYMPHOMA_DN
C2	KLEIN_PRIMARY_EFFUSION_LYMPHOMA_UP
C2	KLEIN_TARGETS_OF_BCR_ABL1_FUSION
C2	LU_IL4_SIGNALING
C2	MATTIOLI_MGUS_VS_PCL
C2	MATTIOLI_MULTIPLE_MYELOMA_SUBGROUPS
C2	MOREAUX_B_LYMPHOCYTE_MATURATION_BY_TACI_DN
C2	MOREAUX_B_LYMPHOCYTE_MATURATION_BY_TACI_UP
C2	MUNSHI_MULTIPLE_MYELOMA_DN
C2	MUNSHI_MULTIPLE_MYELOMA_UP
C2	ODONNELL_TARGETS_OF_MYC_AND_TFRC_DN
C2	ODONNELL_TARGETS_OF_MYC_AND_TFRC_UP
C2	ODONNELL_TFRC_TARGETS_DN
C2	ODONNELL_TFRC_TARGETS_UP

C2	PELLICCIOTTA_HDAC_IN_ANTIGEN_PRESENTATION_DN
C2	PELLICCIOTTA_HDAC_IN_ANTIGEN_PRESENTATION_UP
C2	PENG_GLUCOSE_DEPRIVATION_DN
C2	PENG_GLUCOSE_DEPRIVATION_UP
C2	PENG_GLUTAMINE_DEPRIVATION_DN
C2	PENG_GLUTAMINE_DEPRIVATION_UP
C2	PENG_LEUCINE_DEPRIVATION_DN
C2	PENG_LEUCINE_DEPRIVATION_UP
C2	PENG_RAPAMYCIN_RESPONSE_DN
C2	PENG_RAPAMYCIN_RESPONSE_UP
C2	PID_BCR_5PATHWAY
C2	PID_CASPASE_PATHWAY
C2	PID_MYC_ACTIV_PATHWAY
C2	PID_MYC_PATHWAY
C2	PID_MYC_REPRESS_PATHWAY
C2	PID_NFAT_3PATHWAY
C2	PID_NFAT_TFPATHWAY
C2	REACTOME_ACTIVATION_OF_NF_KAPPAB_IN_B_CELLS
C2	REACTOME_ANTIGEN_ACTIVATES_B_CELL_RECEPTOR_LEADING_TO_GENERATION_OF_SECOND_MESSENGERS
C2	REACTOME_APC_C_CDC20_MEDIATED_DEGRADATION_OF_MITOTIC_PROTEINS
C2	REACTOME_APC_C_CDH1_MEDIATED_DEGRADATION_OF_CDC20_AND_OTHER_APC_C_CDH1_TARGETED_PROTEINS_IN_LATE_MITOSIS_EARLY_G1
C2	REACTOME_APOPTOSIS
C2	REACTOME_APOPTOSIS_INDUCED_DNA_FRAGMENTATION
C2	REACTOME_APOPTOTIC_CLEAVAGE_OF_CELL_ADHESION_PROTEINS
C2	REACTOME_APOPTOTIC_CLEAVAGE_OF_CELLULAR_PROTEINS
C2	REACTOME_APOPTOTIC_EXECUTION_PHASE
C2	REACTOME_CELL_CYCLE
C2	REACTOME_CELL_CYCLE_CHECKPOINTS
C2	REACTOME_CELL_CYCLE_MITOTIC
C2	REACTOME_DOWNSTREAM_SIGNALING_EVENTS_OF_B_CELL_RECEPTOR_BCR
C2	REACTOME_EXTRINSIC_PATHWAY_FOR_APOPTOSIS
C2	REACTOME_INHIBITION_OF_THE_PROTEOLYTIC_ACTIVITY_OF_APC_C_REQUIRED_FOR_THE_ONSET_OF_ANAPHASE_BY_MITOTIC_SPINDLE_CHECKPOINT_COMPONENTS
C2	REACTOME_INTRINSIC_PATHWAY_FOR_APOPTOSIS

C2	REACTOME_LOSS_OF_NLP_FROM_MITOTIC_CENTROSOMES
C2	REACTOME_MITOTIC_G1_G1_S_PHASES
C2	REACTOME_MITOTIC_G2_G2_M_PHASES
C2	REACTOME_MITOTIC_M_M_G1_PHASES
C2	REACTOME_MITOTIC_PROMETAPHASE
C2	REACTOME_RECRUITMENT_OF_MITOTIC_CENTROSOME_PROTEINS_AND_COMPLEXES
C2	REACTOME_RECRUITMENT_OF_NUMA_TO_MITOTIC_CENTROSOMES
C2	REACTOME_REGULATION_OF_APOPTOSIS
C2	REACTOME_REGULATION_OF_MITOTIC_CELL_CYCLE
C2	REACTOME_ROLE_OF_DCC_IN_REGULATING_APOPTOSIS
C2	REACTOME_SIGNALING_BY_THE_B_CELL_RECEPTOR_BCR
C2	SA_B_CELL_RECEPTOR_COMPLEXES
C2	SA_CASPASE_CASCADE
C2	SA_FAS_SIGNALING
C2	SA_PROGRAMMED_CELL_DEATH
C2	SA_REG_CASCADE_OF_CYCLIN_EXPR
C2	SCHLOSSER_MYC_AND_SERUM_RESPONSE_SYNERGY
C2	SCHLOSSER_MYC_TARGETS_AND_SERUM_RESPONSE_DN
C2	SCHLOSSER_MYC_TARGETS_AND_SERUM_RESPONSE_UP
C2	SCHLOSSER_MYC_TARGETS_REPRESSED_BY_SERUM
C2	SCHLOSSER_SERUM_RESPONSE_AUGMENTED_BY_MYC
C2	SCHLOSSER_SERUM_RESPONSE_DN
C2	SCHLOSSER_SERUM_RESPONSE_UP
C2	SCHUHMACHER_MYC_TARGETS_DN
C2	SCHUHMACHER_MYC_TARGETS_UP
C2	SHAFFER_IRF4_TARGETS_IN_ACTIVATED_B_LYMPHOCYTE
C2	SHAFFER_IRF4_TARGETS_IN_MYELOMA_VS_MATURE_B_LYMPHOCYTE
C2	SHAFFER_IRF4_TARGETS_IN_PLASMA_CELL_VS_MATURE_B_LYMPHOCYTE
C2	SHIPP_DLBCL_CURED_VS_FATAL_DN
C2	SHIPP_DLBCL_CURED_VS_FATAL_UP
C2	SHIPP_DLBCL_VS_FOLLICULAR_LYMPHOMA_DN
C2	SHIPP_DLBCL_VS_FOLLICULAR_LYMPHOMA_UP
C2	SIG_BCR_SIGNALING_PATHWAY
C2	SIG_IL4RECEPTOR_IN_B_LYPHOCYTES
C2	SIG_PIP3_SIGNALING_IN_B_LYMPHOCYTES
C2	SMIRNOV_RESPONSE_TO_IR_2HR_DN

C2	SMIRNOV_RESPONSE_TO_IR_2HR_UP
C2	SMIRNOV_RESPONSE_TO_IR_6HR_DN
C2	SMIRNOV_RESPONSE_TO_IR_6HR_UP
C2	ST_B_CELL_ANTIGEN_RECEPTOR
C2	TARTE_PLASMA_CELL_VS_B_LYMPHOCYTE_DN
C2	TARTE_PLASMA_CELL_VS_B_LYMPHOCYTE_UP
C2	TARTE_PLASMA_CELL_VS_PLASMABLAST_DN
C2	TARTE_PLASMA_CELL_VS_PLASMABLAST_UP
C2	TAVOR_CEBPA_TARGETS_DN
C2	TAVOR_CEBPA_TARGETS_UP
C2	TRACEY_RESISTANCE_TO_IFNA2_DN
C2	TRACEY_RESISTANCE_TO_IFNA2_UP
C2	WAGNER_APO2_SENSITIVITY
C2	WHITFIELD_CELL_CYCLE_LITERATURE
C2	ZHAN_EARLY_DIFFERENTIATION_GENES_DN
C2	ZHAN_EARLY_DIFFERENTIATION_GENES_UP
C2	ZHAN_LATE_DIFFERENTIATION_GENES_DN
C2	ZHAN_LATE_DIFFERENTIATION_GENES_UP
C2	ZHAN_MULTIPLE_MYELOMA_DN
C2	ZHAN_MULTIPLE_MYELOMA_PR_DN
C2	ZHAN_MULTIPLE_MYELOMA_PR_UP
C2	ZHAN_MULTIPLE_MYELOMA_SPIKED
C2	ZHAN_MULTIPLE_MYELOMA_UP
C2	ZHAN_V1_LATE_DIFFERENTIATION_GENES_DN
C2	ZHAN_V1_LATE_DIFFERENTIATION_GENES_UP
C2	ZHAN_V2_LATE_DIFFERENTIATION_GENES
C2	ZHAN_VARIABLE_EARLY_DIFFERENTIATION_GENES_DN
C2	ZHAN_VARIABLE_EARLY_DIFFERENTIATION_GENES_UP
C3	CACGTG_MYC_Q2
C3	MYC_Q2
C3	NFAT_Q4_01
C3	NFAT_Q6
C3	TGGAAA_NFAT_Q4_01
C4	MODULE_126
C4	MODULE_291
C4	MODULE_312

C4	MODULE_358
C4	MODULE_361
C4	MODULE_456
C4	MODULE_537
C4	MODULE_54
C4	MODULE_56
C4	MODULE_573
C4	MORF_MYC
C5	GO_ACTIVATION_OF_CYSTEINE_TYPE_ENDOPEPTIDASE_ACTIVITY_INVOLVED_IN_APOPTOTIC_SIGNALING_PATHWAY
C5	GO_ANAPHASE_PROMOTING_COMPLEX
C5	GO_APOPTOTIC_CELL_CLEARANCE
C5	GO_APOPTOTIC_DNA_FRAGMENTATION
C5	GO_APOPTOTIC_MITOCHONDRIAL_CHANGES
C5	GO_APOPTOTIC_NUCLEAR_CHANGES
C5	GO_APOPTOTIC_PROCESS_INVOLVED_IN_DEVELOPMENT
C5	GO_APOPTOTIC_PROCESS_INVOLVED_IN_MORPHOGENESIS
C5	GO_APOPTOTIC_SIGNALING_PATHWAY
C5	GO_B_CELL_ACTIVATION
C5	GO_B_CELL_ACTIVATION_INVOLVED_IN_IMMUNE_RESPONSE
C5	GO_B_CELL_DIFFERENTIATION
C5	GO_B_CELL_HOMEOSTASIS
C5	GO_B_CELL_MEDIATED_IMMUNITY
C5	GO_B_CELL_PROLIFERATION
C5	GO_B_CELL_RECEPTOR_SIGNALING_PATHWAY
C5	GO_CELL_AGING
C5	GO_CELL_CYCLE
C5	GO_CELL_CYCLE_ARREST
C5	GO_CELL_CYCLE_CHECKPOINT
C5	GO_CELL_CYCLE_DNA_REPLICATION
C5	GO_CELL_CYCLE_G1_S_PHASE_TRANSITION
C5	GO_CELL_CYCLE_G2_M_PHASE_TRANSITION
C5	GO_CELL_CYCLE_PHASE_TRANSITION
C5	GO_CELL_CYCLE_PROCESS
C5	GO_CELL_DEATH
C5	GO_CELL_DIVISION_SITE

C5	GO_CELLULAR_COMPONENT_DISASSEMBLY_INVOLVED_IN_EXECUTION_PHASE_OF_APOPTOSIS
C5	GO_CELLULAR_SENESCENCE
C5	GO_CENTRIOLE
C5	GO_CENTROSOME_CYCLE
C5	GO_CHROMOSOME_CONDENSATION
C5	GO_CHROMOSOME_SEPARATION
C5	GO_CYCLIN_BINDING
C5	GO_CYSTEINE_TYPE_ENDOPEPTIDASE_ACTIVITY_INVOLVED_IN_APOPTOTIC_PROCESS
C5	GO_CYSTEINE_TYPE_ENDOPEPTIDASE_INHIBITOR_ACTIVITY_INVOLVED_IN_APOPTOTIC_PROCESS
C5	GO_CYSTEINE_TYPE_ENDOPEPTIDASE_REGULATOR_ACTIVITY_INVOLVED_IN_APOPTOTIC_PROCESS
C5	GO_CYTOPLASMIC_DYNEIN_COMPLEX
C5	GO_DEATH_RECEPTOR_ACTIVITY
C5	GO_DNA_INTEGRITY_CHECKPOINT
C5	GO_DNA_REPLICATION_CHECKPOINT
C5	GO_ESTABLISHMENT_OF_MITOTIC_SPINDLE_LOCALIZATION
C5	GO_ESTABLISHMENT_OF_MITOTIC_SPINDLE_ORIENTATION
C5	GO_EXECUTION_PHASE_OF_APOPTOSIS
C5	GO_EXIT_FROM_MITOSIS
C5	GO_EXTRINSIC_APOPTOTIC_SIGNALING_PATHWAY
C5	GO_EXTRINSIC_APOPTOTIC_SIGNALING_PATHWAY_VIA_DEATH_DOMAIN_RECEPTORS
C5	GO_G1_DNA_DAMAGE_CHECKPOINT
C5	GO_G2_DNA_DAMAGE_CHECKPOINT
C5	GO_HOMOLOGOUS_CHROMOSOME_SEGREGATION
C5	GO_INFLAMMATORY_RESPONSE_TO_ANTIGENIC_STIMULUS
C5	GO_INTRA_S_DNA_DAMAGE_CHECKPOINT
C5	GO_INTRINSIC_APOPTOTIC_SIGNALING_PATHWAY
C5	GO_INTRINSIC_APOPTOTIC_SIGNALING_PATHWAY_BY_P53_CLASS_MEDIATOR
C5	GO_INTRINSIC_APOPTOTIC_SIGNALING_PATHWAY_IN_RESPONSE_TO_DNA_DAMAGE
C5	GO_INTRINSIC_APOPTOTIC_SIGNALING_PATHWAY_IN_RESPONSE_TO_DNA_DAMAGE_BY_P53_CLASS_MEDIATOR
C5	GO_INTRINSIC_APOPTOTIC_SIGNALING_PATHWAY_IN_RESPONSE_TO_ENDOPLASMIC_RETICULUM_STRESS
C5	GO_ISOTYPE_SWITCHING
C5	GO_LEUKOCYTE_APOPTOTIC_PROCESS
C5	GO_LEUKOCYTE_PROLIFERATION
C5	GO_LYMPHOCYTE_APOPTOTIC_PROCESS
C5	GO_LYMPHOCYTE_HOMEOSTASIS

C5	GO_MATURE_B_CELL_DIFFERENTIATION
C5	GO_MATURE_B_CELL_DIFFERENTIATION_INVOLVED_IN_IMMUNE_RESPONSE
C5	GO_MITOTIC_CELL_CYCLE
C5	GO_MITOTIC_CELL_CYCLE_ARREST
C5	GO_MITOTIC_CELL_CYCLE_CHECKPOINT
C5	GO_MITOTIC_CHROMOSOME_CONDENSATION
C5	GO_MITOTIC_CYTOKINESIS
C5	GO_MITOTIC_DNA_INTEGRITY_CHECKPOINT
C5	GO_MITOTIC_G2_DNA_DAMAGE_CHECKPOINT
C5	GO_MITOTIC_G2_M_TRANSITION_CHECKPOINT
C5	GO_MITOTIC_NUCLEAR_DIVISION
C5	GO_MITOTIC_RECOMBINATION
C5	GO_MITOTIC_SISTER_CHROMATID_COHESION
C5	GO_MITOTIC_SISTER_CHROMATID_SEGREGATION
C5	GO_MITOTIC_SPINDLE
C5	GO_MITOTIC_SPINDLE_ASSEMBLY
C5	GO_MITOTIC_SPINDLE_ORGANIZATION
C5	GO_NECROTIC_CELL_DEATH
C5	GO_NEGATIVE_REGULATION_OF_APOPTOTIC_SIGNALING_PATHWAY
C5	GO_NEGATIVE_REGULATION_OF_B_CELL_ACTIVATION
C5	GO_NEGATIVE_REGULATION_OF_B_CELL_APOPTOTIC_PROCESS
C5	GO_NEGATIVE_REGULATION_OF_B_CELL_PROLIFERATION
C5	GO_NEGATIVE_REGULATION_OF_CELL_CYCLE
C5	GO_NEGATIVE_REGULATION_OF_CELL_CYCLE_ARREST
C5	GO_NEGATIVE_REGULATION_OF_CELL_CYCLE_G1_S_PHASE_TRANSITION
C5	GO_NEGATIVE_REGULATION_OF_CELL_CYCLE_G2_M_PHASE_TRANSITION
C5	GO_NEGATIVE_REGULATION_OF_CELL_CYCLE_PHASE_TRANSITION
C5	GO_NEGATIVE_REGULATION_OF_CELL_CYCLE_PROCESS
C5	GO_NEGATIVE_REGULATION_OF_DNA_DAMAGE_RESPONSE_SIGNAL_TRANSDUCTION_BY_P53_CLASS_MEDIATOR
C5	GO_NEGATIVE_REGULATION_OF_ENDOPLASMIC_RETICULUM_STRESS_INDUCED_INTRINSIC_APOPTOTIC_SIGNALING_PATHWAY
C5	GO_NEGATIVE_REGULATION_OF_ENDOTHELIAL_CELL_APOPTOTIC_PROCESS
C5	GO_NEGATIVE_REGULATION_OF_EPITHELIAL_CELL_APOPTOTIC_PROCESS
C5	GO_NEGATIVE_REGULATION_OF_EXTRINSIC_APOPTOTIC_SIGNALING_PATHWAY
C5	GO_NEGATIVE_REGULATION_OF_EXTRINSIC_APOPTOTIC_SIGNALING_PATHWAY_VIA_DEATH_DOMAIN_RECEPT

	ORS
C5	GO_NEGATIVE_REGULATION_OF_INTRINSIC_APOPTOTIC_SIGNALING_PATHWAY
C5	GO_NEGATIVE_REGULATION_OF_INTRINSIC_APOPTOTIC_SIGNALING_PATHWAY_BY_P53_CLASS_MEDIATOR
C5	GO_NEGATIVE_REGULATION_OF_INTRINSIC_APOPTOTIC_SIGNALING_PATHWAY_IN_RESPONSE_TO_DNA_DAMAGE
C5	GO_NEGATIVE_REGULATION_OF_LEUKOCYTE_APOPTOTIC_PROCESS
C5	GO_NEGATIVE_REGULATION_OF_LEUKOCYTE_PROLIFERATION
C5	GO_NEGATIVE_REGULATION_OF_LYMPHOCYTE_APOPTOTIC_PROCESS
C5	GO_NEGATIVE_REGULATION_OF_MITOTIC_CELL_CYCLE
C5	GO_NEGATIVE_REGULATION_OF_MITOTIC_NUCLEAR_DIVISION
C5	GO_NEGATIVE_REGULATION_OF_OXIDATIVE_STRESS_INDUCED_INTRINSIC_APOPTOTIC_SIGNALING_PATHWAY
C5	GO_NEGATIVE_REGULATION_OF_RELEASE_OF_CYTOCHROME_C_FROM_MITOCHONDRIA
C5	GO_NUCLEAR_CHROMOSOME
C5	GO_NUCLEOLAR_PART
C5	GO_NUCLEOLUS
C5	GO_PEPTIDASE_ACTIVATOR_ACTIVITY_INVOLVED_IN_APOPTOTIC_PROCESS
C5	GO_POSITIVE_REGULATION_OF_APOPTOTIC_SIGNALING_PATHWAY
C5	GO_POSITIVE_REGULATION_OF_B_CELL_ACTIVATION
C5	GO_POSITIVE_REGULATION_OF_B_CELL_DIFFERENTIATION
C5	GO_POSITIVE_REGULATION_OF_B_CELL_MEDIATED_IMMUNITY
C5	GO_POSITIVE_REGULATION_OF_B_CELL_PROLIFERATION
C5	GO_POSITIVE_REGULATION_OF_CELL_CYCLE
C5	GO_POSITIVE_REGULATION_OF_CELL_CYCLE_ARREST
C5	GO_POSITIVE_REGULATION_OF_CELL_CYCLE_G1_S_PHASE_TRANSITION
C5	GO_POSITIVE_REGULATION_OF_CELL_CYCLE_G2_M_PHASE_TRANSITION
C5	GO_POSITIVE_REGULATION_OF_CELL_CYCLE_PHASE_TRANSITION
C5	GO_POSITIVE_REGULATION_OF_CELL_CYCLE_PROCESS
C5	GO_POSITIVE_REGULATION_OF_CYSTEINE_TYPE_ENDOPEPTIDASE_ACTIVITY_INVOLVED_IN_APOPTOTIC_SIGNALING_PATHWAY
C5	GO_POSITIVE_REGULATION_OF_DNA_DAMAGE_RESPONSE_SIGNAL_TRANSDUCTION_BY_P53_CLASS_MEDIATOR
C5	GO_POSITIVE_REGULATION_OF_EXECUTION_PHASE_OF_APOPTOSIS
C5	GO_POSITIVE_REGULATION_OF_EXTRINSIC_APOPTOTIC_SIGNALING_PATHWAY
C5	GO_POSITIVE_REGULATION_OF_EXTRINSIC_APOPTOTIC_SIGNALING_PATHWAY_IN_ABSENCE_OF_LIGAND
C5	GO_POSITIVE_REGULATION_OF_EXTRINSIC_APOPTOTIC_SIGNALING_PATHWAY_VIA_DEATH_DOMAIN_RECEPTORS
C5	GO_POSITIVE_REGULATION_OF_G1_S_TRANSITION_OF_MITOTIC_CELL_CYCLE

C5	GO_POSITIVE_REGULATION_OF_INTRINSIC_APOPTOTIC_SIGNALING_PATHWAY
C5	GO_POSITIVE_REGULATION_OF_LEUKOCYTE_APOPTOTIC_PROCESS
C5	GO_POSITIVE_REGULATION_OF_LEUKOCYTE_PROLIFERATION
C5	GO_POSITIVE_REGULATION_OF_LYMPHOCYTE_APOPTOTIC_PROCESS
C5	GO_POSITIVE_REGULATION_OF_MITOCHONDRIAL_OUTER_MEMBRANE_PERMEABILIZATION_INVOLVED_IN_APOPTOTIC_SIGNALING_PATHWAY
C5	GO_POSITIVE_REGULATION_OF_MITOTIC_CELL_CYCLE
C5	GO_POSITIVE_REGULATION_OF_MITOTIC_NUCLEAR_DIVISION
C5	GO_POSITIVE_REGULATION_OF_MITOTIC_SISTER_CHROMATID_SEPARATION
C5	GO_POSITIVE_REGULATION_OF_NFAT_PROTEIN_IMPORT_INTO_NUCLEUS
C5	GO_POSITIVE_REGULATION_OF_RELEASE_OF_CYTOCHROME_C_FROM_MITOCHONDRIA
C5	GO_PROTEIN_K11_LINKED_UBIQUITINATION
C5	GO_RAN_GTPASE_BINDING
C5	GO_REGULATION_OF_APOPTOTIC_SIGNALING_PATHWAY
C5	GO_REGULATION_OF_B_CELL_ACTIVATION
C5	GO_REGULATION_OF_B_CELL_APOPTOTIC_PROCESS
C5	GO_REGULATION_OF_B_CELL_DIFFERENTIATION
C5	GO_REGULATION_OF_B_CELL_MEDIATED_IMMUNITY
C5	GO_REGULATION_OF_B_CELL_PROLIFERATION
C5	GO_REGULATION_OF_B_CELL_RECEPTOR_SIGNALING_PATHWAY
C5	GO_REGULATION_OF_CALCINEURIN_NFAT_SIGNALING_CASCADE
C5	GO_REGULATION_OF_CELL_CYCLE
C5	GO_REGULATION_OF_CELL_CYCLE_ARREST
C5	GO_REGULATION_OF_CELL_CYCLE_CHECKPOINT
C5	GO_REGULATION_OF_CELL_CYCLE_G1_S_PHASE_TRANSITION
C5	GO_REGULATION_OF_CELL_CYCLE_G2_M_PHASE_TRANSITION
C5	GO_REGULATION_OF_CELL_CYCLE_PHASE_TRANSITION
C5	GO_REGULATION_OF_CELL_CYCLE_PROCESS
C5	GO_REGULATION_OF_CYSTEINE_TYPE_ENDOPEPTIDASE_ACTIVITY_INVOLVED_IN_APOPTOTIC_SIGNALING_PATHWAY
C5	GO_REGULATION_OF_DNA_DAMAGE_RESPONSE_SIGNAL_TRANSDUCTION_BY_P53_CLASS_MEDIATOR
C5	GO_REGULATION_OF_ENDOPLASMIC_RETICULUM_STRESS_INDUCED_INTRINSIC_APOPTOTIC_SIGNALING_PATHWAY
C5	GO_REGULATION_OF_EXECUTION_PHASE_OF_APOPTOSIS
C5	GO_REGULATION_OF_EXIT_FROM_MITOSIS
C5	GO_REGULATION_OF_EXTRINSIC_APOPTOTIC_SIGNALING_PATHWAY

C5	GO_REGULATION_OF_EXTRINSIC_APOPTOTIC_SIGNALING_PATHWAY_IN_ABSENCE_OF_LIGAND
C5	GO_REGULATION_OF_EXTRINSIC_APOPTOTIC_SIGNALING_PATHWAY_VIA_DEATH_DOMAIN_RECEPTORS
C5	GO_REGULATION_OF_INTRINSIC_APOPTOTIC_SIGNALING_PATHWAY
C5	GO_REGULATION_OF_INTRINSIC_APOPTOTIC_SIGNALING_PATHWAY_BY_P53_CLASS_MEDIATOR
C5	GO_REGULATION_OF_INTRINSIC_APOPTOTIC_SIGNALING_PATHWAY_IN_RESPONSE_TO_DNA_DAMAGE
C5	GO_REGULATION_OF_INTRINSIC_APOPTOTIC_SIGNALING_PATHWAY_IN_RESPONSE_TO_DNA_DAMAGE_BY_P53_CLASS_MEDIATOR
C5	GO_REGULATION_OF_LEUKOCYTE_APOPTOTIC_PROCESS
C5	GO_REGULATION_OF_LEUKOCYTE_PROLIFERATION
C5	GO_REGULATION_OF_LYMPHOCYTE_APOPTOTIC_PROCESS
C5	GO_REGULATION_OF_MITOCHONDRIAL_MEMBRANE_PERMEABILITY_INVOLVED_IN_APOPTOTIC_PROCESS
C5	GO_REGULATION_OF_MITOCHONDRIAL_OUTER_MEMBRANE_PERMEABILIZATION_INVOLVED_IN_APOPTOTIC_SIGNALING_PATHWAY
C5	GO_REGULATION_OF_MITOTIC_CELL_CYCLE
C5	GO_REGULATION_OF_MITOTIC_SPINDLE_CHECKPOINT
C5	GO_REGULATION_OF_NFAT_PROTEIN_IMPORT_INTO_NUCLEUS
C5	GO_REGULATION_OF_NUCLEAR_CELL_CYCLE_DNA_REPLICATION
C5	GO_REGULATION_OF_OXIDATIVE_STRESS_INDUCED_INTRINSIC_APOPTOTIC_SIGNALING_PATHWAY
C5	GO_REGULATION_OF_PROTEIN_INSERTION_INTO_MITOCHONDRIAL_MEMBRANE_INVOLVED_IN_APOPTOTIC_SIGNALING_PATHWAY
C5	GO_REGULATION_OF_RELEASE_OF_CYTOCHROME_C_FROM_MITOCHONDRIA
C5	GO_REGULATION_OF_SPINDLE_CHECKPOINT
C5	GO_REGULATION_OF_TRANSCRIPTION_INVOLVED_IN_G1_S_TRANSITION_OF_MITOTIC_CELL_CYCLE
C5	GO_REGULATION_OF_TUMOR_NECROSIS_FACTOR_BIOSYNTHETIC_PROCESS
C5	GO_RELEASE_OF_CYTOCHROME_C_FROM_MITOCHONDRIA
C5	GO_SIGNAL_TRANSDUCTION_BY_P53_CLASS_MEDIATOR
C5	GO_SISTER_CHROMATID_COHESION
C5	GO_SISTER_CHROMATID_SEGREGATION
C5	GO_SPINDLE
C5	GO_SPINDLE_CHECKPOINT
C5	GO_SPINDLE_MICROTUBULE
C7	GSE10325_BCELL_VS_LUPUS_BCELL_DN
C7	GSE10325_BCELL_VS_LUPUS_BCELL_UP
C7	GSE10325_BCELL_VS_MYELOID_DN
C7	GSE10325_BCELL_VS_MYELOID_UP
C7	GSE10325_CD4_TCELL_VS_BCELL_DN

C7	GSE10325_CD4_TCELL_VS_BCELL_UP
C7	GSE10325_LUPUS_BCELL_VS_LUPUS_MYELOID_DN
C7	GSE10325_LUPUS_BCELL_VS_LUPUS_MYELOID_UP
C7	GSE10325_LUPUS_CD4_TCELL_VS_LUPUS_BCELL_DN
C7	GSE10325_LUPUS_CD4_TCELL_VS_LUPUS_BCELL_UP
C7	GSE12366_GC_BCELL_VS_PLASMA_CELL_DN
C7	GSE12366_GC_BCELL_VS_PLASMA_CELL_UP
C7	GSE12366_GC_VS_MEMORY_BCELL_DN
C7	GSE12366_GC_VS_MEMORY_BCELL_UP
C7	GSE12366_GC_VS_NAIVE_BCELL_DN
C7	GSE12366_GC_VS_NAIVE_BCELL_UP
C7	GSE12366_NAIVE_VS_MEMORY_BCELL_DN
C7	GSE12366_NAIVE_VS_MEMORY_BCELL_UP
C7	GSE12366_PLASMA_CELL_VS_MEMORY_BCELL_DN
C7	GSE12366_PLASMA_CELL_VS_MEMORY_BCELL_UP
C7	GSE12366_PLASMA_CELL_VS_NAIVE_BCELL_DN
C7	GSE12366_PLASMA_CELL_VS_NAIVE_BCELL_UP
C7	GSE12845_IGD_NEG_BLOOD_VS_DARKZONE_GC_TONSIL_BCELL_DN
C7	GSE12845_IGD_NEG_BLOOD_VS_DARKZONE_GC_TONSIL_BCELL_UP
C7	GSE12845_IGD_NEG_BLOOD_VS_NAIVE_TONSIL_BCELL_DN
C7	GSE12845_IGD_NEG_BLOOD_VS_NAIVE_TONSIL_BCELL_UP
C7	GSE12845_IGD_NEG_BLOOD_VS_PRE_GC_TONSIL_BCELL_DN
C7	GSE12845_IGD_NEG_BLOOD_VS_PRE_GC_TONSIL_BCELL_UP
C7	GSE12845_IGD_POS_BLOOD_VS_DARKZONE_GC_TONSIL_BCELL_DN
C7	GSE12845_IGD_POS_BLOOD_VS_DARKZONE_GC_TONSIL_BCELL_UP
C7	GSE12845_IGD_POS_BLOOD_VS_NAIVE_TONSIL_BCELL_DN
C7	GSE12845_IGD_POS_BLOOD_VS_NAIVE_TONSIL_BCELL_UP
C7	GSE12845_IGD_POS_BLOOD_VS_PRE_GC_TONSIL_BCELL_DN
C7	GSE12845_IGD_POS_BLOOD_VS_PRE_GC_TONSIL_BCELL_UP
C7	GSE12845_IGD_POS_VS_NEG_BLOOD_BCELL_DN
C7	GSE12845_IGD_POS_VS_NEG_BLOOD_BCELL_UP
C7	GSE12845_NAIVE_VS_DARKZONE_GC_TONSIL_BCELL_DN
C7	GSE12845_NAIVE_VS_DARKZONE_GC_TONSIL_BCELL_UP
C7	GSE12845_NAIVE_VS_PRE_GC_TONSIL_BCELL_DN
C7	GSE12845_NAIVE_VS_PRE_GC_TONSIL_BCELL_UP
C7	GSE12845_PRE_GC_VS_DARKZONE_GC_TONSIL_BCELL_DN

C7	GSE12845_PRE_GC_VS_DARKZONE_GC_TONSIL_BCELL_UP
C7	GSE13411_IGM_MEMORY_BCELL_VS_PLASMA_CELL_DN
C7	GSE13411_IGM_MEMORY_BCELL_VS_PLASMA_CELL_UP
C7	GSE13411_IGM_VS_SWITCHED_MEMORY_BCELL_DN
C7	GSE13411_IGM_VS_SWITCHED_MEMORY_BCELL_UP
C7	GSE13411_NAIVE_BCELL_VS_PLASMA_CELL_DN
C7	GSE13411_NAIVE_BCELL_VS_PLASMA_CELL_UP
C7	GSE13411_NAIVE_VS_IGM_MEMORY_BCELL_DN
C7	GSE13411_NAIVE_VS_IGM_MEMORY_BCELL_UP
C7	GSE13411_NAIVE_VS_MEMORY_BCELL_DN
C7	GSE13411_NAIVE_VS_MEMORY_BCELL_UP
C7	GSE13411_NAIVE_VS_SWITCHED_MEMORY_BCELL_DN
C7	GSE13411_NAIVE_VS_SWITCHED_MEMORY_BCELL_UP
C7	GSE13411_PLASMA_CELL_VS_MEMORY_BCELL_DN
C7	GSE13411_PLASMA_CELL_VS_MEMORY_BCELL_UP
C7	GSE13411_SWITCHED_MEMORY_BCELL_VS_PLASMA_CELL_DN
C7	GSE13411_SWITCHED_MEMORY_BCELL_VS_PLASMA_CELL_UP
C7	GSE15271_CXCR4_POS_VS_NEG_GC_BCELL_DN
C7	GSE15271_CXCR4_POS_VS_NEG_GC_BCELL_UP
C7	GSE17186_BLOOD_VS_CORD_BLOOD_CD21HIGH_TRANSITIONAL_BCELL_DN
C7	GSE17186_BLOOD_VS_CORD_BLOOD_CD21HIGH_TRANSITIONAL_BCELL_UP
C7	GSE17186_BLOOD_VS_CORD_BLOOD_CD21LOW_TRANSITIONAL_BCELL_DN
C7	GSE17186_BLOOD_VS_CORD_BLOOD_CD21LOW_TRANSITIONAL_BCELL_UP
C7	GSE17186_BLOOD_VS_CORD_BLOOD_NAIVE_BCELL_DN
C7	GSE17186_BLOOD_VS_CORD_BLOOD_NAIVE_BCELL_UP
C7	GSE17186_CD21LOW_VS_CD21HIGH_TRANSITIONAL_BCELL_CORD_BLOOD_DN
C7	GSE17186_CD21LOW_VS_CD21HIGH_TRANSITIONAL_BCELL_CORD_BLOOD_UP
C7	GSE17186_CD21LOW_VS_CD21HIGH_TRANSITIONAL_BCELL_DN
C7	GSE17186_CD21LOW_VS_CD21HIGH_TRANSITIONAL_BCELL_UP
C7	GSE17186_MEMORY_VS_CD21HIGH_TRANSITIONAL_BCELL_DN
C7	GSE17186_MEMORY_VS_CD21HIGH_TRANSITIONAL_BCELL_UP
C7	GSE17186_MEMORY_VS_CD21LOW_TRANSITIONAL_BCELL_DN
C7	GSE17186_MEMORY_VS_CD21LOW_TRANSITIONAL_BCELL_UP
C7	GSE17186_MEMORY_VS_NAIVE_BCELL_DN
C7	GSE17186_MEMORY_VS_NAIVE_BCELL_UP
C7	GSE17186_NAIVE_VS_CD21HIGH_TRANSITIONAL_BCELL_CORD_BLOOD_DN

C7	GSE17186_NAIVE_VS_CD21HIGH_TRANSITIONAL_BCELL_CORD_BLOOD_UP
C7	GSE17186_NAIVE_VS_CD21HIGH_TRANSITIONAL_BCELL_DN
C7	GSE17186_NAIVE_VS_CD21HIGH_TRANSITIONAL_BCELL_UP
C7	GSE17186_NAIVE_VS_CD21LOW_TRANSITIONAL_BCELL_CORD_BLOOD_DN
C7	GSE17186_NAIVE_VS_CD21LOW_TRANSITIONAL_BCELL_CORD_BLOOD_UP
C7	GSE17186_NAIVE_VS_CD21LOW_TRANSITIONAL_BCELL_DN
C7	GSE17186_NAIVE_VS_CD21LOW_TRANSITIONAL_BCELL_UP
C7	GSE22229_RENAL_TRANSPLANT_IMMUNOSUPP_THERAPY_VS_HEALTHY_PBMC_DN
C7	GSE22229_RENAL_TRANSPLANT_IMMUNOSUPP_THERAPY_VS_HEALTHY_PBMC_UP
C7	GSE22229_RENAL_TRANSPLANT_VS_HEALTHY_PBMC_DN
C7	GSE22229_RENAL_TRANSPLANT_VS_HEALTHY_PBMC_UP
C7	GSE22229_UNTREATED_VS_IMMUNOSUPP_THERAPY_RENAL_TRANSPLANT_PATIENT_PBMC_DN
C7	GSE22229_UNTREATED_VS_IMMUNOSUPP_THERAPY_RENAL_TRANSPLANT_PATIENT_PBMC_UP
C7	GSE22886_CD4_TCELL_VS_BCELL_NAIVE_DN
C7	GSE22886_CD4_TCELL_VS_BCELL_NAIVE_UP
C7	GSE22886_CD8_TCELL_VS_BCELL_NAIVE_DN
C7	GSE22886_CD8_TCELL_VS_BCELL_NAIVE_UP
C7	GSE22886_IGA_VS_IGM_MEMORY_BCELL_DN
C7	GSE22886_IGA_VS_IGM_MEMORY_BCELL_UP
C7	GSE22886_IGG_IGA_MEMORY_BCELL_VS_BLOOD_PLASMA_CELL_DN
C7	GSE22886_IGG_IGA_MEMORY_BCELL_VS_BLOOD_PLASMA_CELL_UP
C7	GSE22886_IGG_IGA_MEMORY_BCELL_VS_BM_PLASMA_CELL_DN
C7	GSE22886_IGG_IGA_MEMORY_BCELL_VS_BM_PLASMA_CELL_UP
C7	GSE22886_IGM_MEMORY_BCELL_VS_BLOOD_PLASMA_CELL_DN
C7	GSE22886_IGM_MEMORY_BCELL_VS_BLOOD_PLASMA_CELL_UP
C7	GSE22886_IGM_MEMORY_BCELL_VS_BM_PLASMA_CELL_DN
C7	GSE22886_IGM_MEMORY_BCELL_VS_BM_PLASMA_CELL_UP
C7	GSE22886_NAIVE_BCELL_VS_BLOOD_PLASMA_CELL_DN
C7	GSE22886_NAIVE_BCELL_VS_BLOOD_PLASMA_CELL_UP
C7	GSE22886_NAIVE_BCELL_VS_BM_PLASMA_CELL_DN
C7	GSE22886_NAIVE_BCELL_VS_BM_PLASMA_CELL_UP
C7	GSE22886_NAIVE_BCELL_VS_DC_DN
C7	GSE22886_NAIVE_BCELL_VS_DC_UP
C7	GSE22886_NAIVE_BCELL_VS_MONOCYTE_DN
C7	GSE22886_NAIVE_BCELL_VS_MONOCYTE_UP
C7	GSE22886_NAIVE_BCELL_VS_NEUTROPHIL_DN

C7	GSE22886_NAIVE_BCELL_VS_NEUTROPHIL_UP
C7	GSE22886_NAIVE_VS_IGG_IGA_MEMORY_BCELL_DN
C7	GSE22886_NAIVE_VS_IGG_IGA_MEMORY_BCELL_UP
C7	GSE22886_NAIVE_VS_IGM_MEMORY_BCELL_DN
C7	GSE22886_NAIVE_VS_IGM_MEMORY_BCELL_UP
C7	GSE22886_TCELL_VS_BCELL_NAIVE_DN
C7	GSE22886_TCELL_VS_BCELL_NAIVE_UP
C7	GSE27670_BLIMP1_VS_LMP1_TRANSDUCED_GC_BCELL_DN
C7	GSE27670_BLIMP1_VS_LMP1_TRANSDUCED_GC_BCELL_UP
C7	GSE27670_CTRL_VS_BLIMP1_TRANSDUCED_GC_BCELL_DN
C7	GSE27670_CTRL_VS_BLIMP1_TRANSDUCED_GC_BCELL_UP
C7	GSE27670_CTRL_VS_LMP1_TRANSDUCED_GC_BCELL_DN
C7	GSE27670_CTRL_VS_LMP1_TRANSDUCED_GC_BCELL_UP
C7	GSE30153_LUPUS_VS_HEALTHY_DONOR_BCELL_DN
C7	GSE30153_LUPUS_VS_HEALTHY_DONOR_BCELL_UP
C7	GSE38697_LIGHT_ZONE_VS_DARK_ZONE_BCELL_DN
C7	GSE38697_LIGHT_ZONE_VS_DARK_ZONE_BCELL_UP
C7	GSE3982_BCELL_VS_BASOPHIL_DN
C7	GSE3982_BCELL_VS_BASOPHIL_UP
C7	GSE3982_BCELL_VS_CENT_MEMORY_CD4_TCELL_DN
C7	GSE3982_BCELL_VS_CENT_MEMORY_CD4_TCELL_UP
C7	GSE3982_BCELL_VS_EFF_MEMORY_CD4_TCELL_DN
C7	GSE3982_BCELL_VS_EFF_MEMORY_CD4_TCELL_UP
C7	GSE3982_BCELL_VS_NKCELL_DN
C7	GSE3982_BCELL_VS_NKCELL_UP
C7	GSE3982_BCELL_VS_TH1_DN
C7	GSE3982_BCELL_VS_TH1_UP
C7	GSE3982_BCELL_VS_TH2_DN
C7	GSE3982_BCELL_VS_TH2_UP
C7	GSE3982_DC_VS_BCELL_DN
C7	GSE3982_DC_VS_BCELL_UP
C7	GSE3982_EOSINOPHIL_VS_BCELL_DN
C7	GSE3982_EOSINOPHIL_VS_BCELL_UP
C7	GSE3982_MAC_VS_BCELL_DN
C7	GSE3982_MAC_VS_BCELL_UP
C7	GSE3982_MAST_CELL_VS_BCELL_DN

C7	GSE3982_MAST_CELL_VS_BCELL_UP
C7	GSE3982_MEMORY_CD4_TCELL_VS_BCELL_DN
C7	GSE3982_MEMORY_CD4_TCELL_VS_BCELL_UP
C7	GSE3982_NEUTROPHIL_VS_BCELL_DN
C7	GSE3982_NEUTROPHIL_VS_BCELL_UP
C7	GSE42724_B1_BCELL_VS_PLASMABLAST_DN
C7	GSE42724_B1_BCELL_VS_PLASMABLAST_UP
C7	GSE42724_MEMORY_BCELL_VS_PLASMABLAST_DN
C7	GSE42724_MEMORY_BCELL_VS_PLASMABLAST_UP
C7	GSE42724_MEMORY_VS_B1_BCELL_DN
C7	GSE42724_MEMORY_VS_B1_BCELL_UP
C7	GSE42724_NAIVE_BCELL_VS_PLASMABLAST_DN
C7	GSE42724_NAIVE_BCELL_VS_PLASMABLAST_UP
C7	GSE42724_NAIVE_VS_B1_BCELL_DN
C7	GSE42724_NAIVE_VS_B1_BCELL_UP
C7	GSE42724_NAIVE_VS_MEMORY_BCELL_DN
C7	GSE42724_NAIVE_VS_MEMORY_BCELL_UP
C7	GSE4590_LARGE_PRE_BCELL_VS_VPREB_POS_LARGE_PRE_BCELL_DN
C7	GSE4590_LARGE_PRE_BCELL_VS_VPREB_POS_LARGE_PRE_BCELL_UP
C7	GSE4590_PRE_BCELL_VS_LARGE_PRE_BCELL_DN
C7	GSE4590_PRE_BCELL_VS_LARGE_PRE_BCELL_UP
C7	GSE4590_PRE_BCELL_VS_SMALL_PRE_BCELL_DN
C7	GSE4590_PRE_BCELL_VS_SMALL_PRE_BCELL_UP
C7	GSE4590_PRE_BCELL_VS_VPREB_POS_LARGE_PRE_BCELL_DN
C7	GSE4590_PRE_BCELL_VS_VPREB_POS_LARGE_PRE_BCELL_UP
C7	GSE4590_SMALL_VS_LARGE_PRE_BCELL_DN
C7	GSE4590_SMALL_VS_LARGE_PRE_BCELL_UP
C7	GSE4590_SMALL_VS_VPREB_POS_LARGE_PRE_BCELL_DN
C7	GSE4590_SMALL_VS_VPREB_POS_LARGE_PRE_BCELL_UP
C7	GSE46143_CTRL_VS_LMP2A_TRANSDUCED_CD10_POS_GC_BCELL_DN
C7	GSE46143_CTRL_VS_LMP2A_TRANSDUCED_CD10_POS_GC_BCELL_UP
H	HALLMARK_ADIPOGENESIS
H	HALLMARK_ALLOGRAFT_REJECTION
H	HALLMARK_ANDROGEN_RESPONSE
H	HALLMARK_ANGIOGENESIS
H	HALLMARK_APICAL_JUNCTION

H	HALLMARK_APICAL_SURFACE
H	HALLMARK_APOPTOSIS
H	HALLMARK_BILE_ACID_METABOLISM
H	HALLMARK_CHOLESTEROL_HOMEOSTASIS
H	HALLMARK_COAGULATION
H	HALLMARK_COMPLEMENT
H	HALLMARK_DNA_REPAIR
H	HALLMARK_E2F_TARGETS
H	HALLMARK_EPITHELIAL_MESENCHYMAL_TRANSITION
H	HALLMARK_ESTROGEN_RESPONSE_EARLY
H	HALLMARK_ESTROGEN_RESPONSE_LATE
H	HALLMARK_FATTY_ACID_METABOLISM
H	HALLMARK_G2M_CHECKPOINT
H	HALLMARK_GLYCOLYSIS
H	HALLMARK_HEDGEHOG_SIGNALING
H	HALLMARK_HEME_METABOLISM
H	HALLMARK_HYPOXIA
H	HALLMARK_IL2_STAT5_SIGNALING
H	HALLMARK_IL6_JAK_STAT3_SIGNALING
H	HALLMARK_INFLAMMATORY_RESPONSE
H	HALLMARK_INTERFERON_ALPHA_RESPONSE
H	HALLMARK_INTERFERON_GAMMA_RESPONSE
H	HALLMARK_KRAS_SIGNALING_DN
H	HALLMARK_KRAS_SIGNALING_UP
H	HALLMARK_MITOTIC_SPINDLE
H	HALLMARK_MTORC1_SIGNALING
H	HALLMARK_MYC_TARGETS_V1
H	HALLMARK_MYC_TARGETS_V2
H	HALLMARK_MYOGENESIS
H	HALLMARK_NOTCH_SIGNALING
H	HALLMARK_OXIDATIVE_PHOSPHORYLATION
H	HALLMARK_P53_PATHWAY
H	HALLMARK_PANCREAS_BETA_CELLS
H	HALLMARK_PEROXISOME
H	HALLMARK_PI3K_AKT_MTOR_SIGNALING
H	HALLMARK_PROTEIN_SECRETION

H	HALLMARK_REACTIVE_OXIGEN_SPECIES_PATHWAY
H	HALLMARK_SPERMATOGENESIS
H	HALLMARK_TGF_BETA_SIGNALING
H	HALLMARK_TNFA_SIGNALING_VIA_NFKB
H	HALLMARK_UNFOLDED_PROTEIN_RESPONSE
H	HALLMARK_UV_RESPONSE_DN
H	HALLMARK_UV_RESPONSE_UP
H	HALLMARK_WNT_BETA_CATENIN_SIGNALING
H	HALLMARK_XENOBIOTIC_METABOLISM
