# Supplementary material for: Immunosuppressive FK506 treatment leads to more frequent EBV-associated lymphoproliferative disease in humanized mice
Source: PLoS Pathog. 2020 Apr 6;16(4):e1008477. doi: 10.1371/journal.ppat.1008477 (PMC7162544; doi:10.1371/journal.ppat.1008477)
Supplement: S2 Table — (PDF) [file ppat.1008477.s003.pdf]

S2 Table. GSEA results.

Set	Comparison	Name	Genes (N=)	Direction	PValue	FDR	PValue Mixed	Deg (N=)	Set Size	FDR Mixed
h	EBV– FK506+vs. EBV– FK506–	HALLMARK_INTERFERON_ALPHA_RESPONSE	92	Down	2.00E-08	7.59E-06	0.003150207	7	97	0.201018672
h	EBV– FK506+vs. EBV– FK506–	HALLMARK_INTERFERON_GAMMA_RESPONSE	182	Down	1.36E-06	0.000275025	0.009262029	8	200	0.338375047
h	EBV– FK506+vs. EBV– FK506–	HALLMARK_MYC_TARGETS_V1	199	Up	1.67E-05	0.001946971	0.311592188	10	200	0.948192912
h	EBV– FK506+vs. EBV– FK506–	HALLMARK_MYC_TARGETS_V2	58	Up	5.63E-05	0.004742164	0.10834336	4	58	0.802430644
h	EBV– FK506+vs. EBV– FK506–	HALLMARK_IL6_JAK_STAT3_SIGNALING	62	Mixed	0.005960181	0.012613351	4.00E-05	1	87	0.012613351
h	EBV– FK506+vs. EBV– FK506–	HALLMARK_HEDGEHOG_SIGNALING	17	Down	0.000259925	0.014198926	0.00875681	0	36	0.331664191
c2	EBV– FK506+vs. EBV– FK506–	HADDAD_B_LYMPHOCYTE_PROGENITOR	254	Down	1.64E-05	0.001946971	0.000733668	6	293	0.111150777
c2	EBV– FK506+vs. EBV– FK506–	DANG_MYC_TARGETS_UP	133	Up	3.00E-05	0.003137071	0.33534322	7	143	0.959158425
c2	EBV– FK506+vs. EBV– FK506–	SA_CASPASE_CASCADE	19	Down	0.000374059	0.019210131	0.029474584	1	19	0.546564493
c2	EBV– FK506+vs. EBV– FK506–	SCHUHMACHER_MYC_TARGETS_UP	79	Up	0.00049091	0.023241509	0.345014275	3	80	0.959158425
c2	EBV– FK506+vs. EBV– FK506–	SCHLOSSER_MYC_TARGETS_AND_SERUM_RESPONSE_DN	46	Up	0.000652493	0.028951521	0.214245524	3	47	0.913287618
c5	EBV– FK506+vs. EBV– FK506–	GO_APOPTOTIC_PROCESS_INVOLVED_IN_DEVELOPMENT	12	Down	0.000922622	0.03630578	0.008687511	0	21	0.331664191
c5	EBV– FK506+vs. EBV– FK506–	GO_NUCLEOLAR_PART	59	Up	0.000947759	0.036816778	0.983340196	5	62	0.999416688
c7	EBV– FK506+vs. EBV– FK506–	GSE42724_NAIVE_BCELL_VS_PLASMABLAST_UP	161	Down	2.92E-10	2.95E-07	1.60E-05	9	199	0.008458777
c7	EBV– FK506+vs. EBV– FK506–	GSE42724_NAIVE_VS_B1_BCELL_DN	167	Up	3.12E-05	0.003152003	0.178167446	5	199	0.892294232
c7	EBV– FK506+vs. EBV– FK506–	GSE3982_BCELL_VS_BASOPHIL_DN	132	Down	0.000178193	0.011487787	0.081341658	4	200	0.727750384
c7	EBV– FK506+vs. EBV– FK506–	GSE22886_CD8_TCELL_VS_BCELL_NAIVE_DN	144	Mixed	0.100428407	0.035942487	0.000154209	3	200	0.035942487
c7	EBV– FK506+vs. EBV– FK506–	GSE3982_BCELL_VS_TH1_DN	147	Down	0.001173728	0.041839955	0.456608282	6	200	0.980075513
c2	EBV+ FK506– vs. EBV– FK506–	PELLICCIOTTA_HDAC_IN_ANTIGEN_PRESENTATION_DN	49	Up	0.000245222	0.014019291	0.307945323	0	49	0.948192912
c7	EBV+ FK506– vs. EBV– FK506–	GSE38697_LIGHT_ZONE_VS_DARK_ZONE_BCELL_DN	32	Down	0.000146512	0.010323981	0.056850591	2	122	0.621587248
c7	EBV+ FK506– vs. EBV– FK506–	GSE10325_LUPUS_BCELL_VS_LUPUS_MYELOID_DN	164	Down	0.001168498	0.041839955	0.273520961	5	200	0.934838348
h	EBV+EBV– FK506+vs. EBV– FK506+	HALLMARK_INTERFERON_ALPHA_RESPONSE	92	Up	9.57E-05	0.007431806	0.09096596	7	97	0.757343626
h	EBV+ FK506+ vs. EBV– FK506+	HALLMARK_IL6_JAK_STAT3_SIGNALING	62	Mixed	0.493932238	0.008458777	1.68E-05	1	87	0.008458777

h	EBV+ FK506+ vs. EBV– FK506+	HALLMARK_MYC_TARGETS_V2	58	Up	0.000409099	0.020659498	0.434204986	4	58	0.978884616
h	EBV+ FK506+ vs. EBV– FK506+	HALLMARK_INTERFERON_GAMMA_RESPONSE	182	Up	0.000771731	0.031629604	0.014856791	8	200	0.389356831
h	EBV+ FK506+ vs. EBV– FK506+	HALLMARK_ALLOGRAFT_REJECTION	164	Mixed	0.007695245	0.043833495	0.000202531	7	200	0.043833495
c2	EBV+ FK506+ vs. EBV– FK506+	PELLICCIOTTA_HDAC_IN_ANTIGEN_PRESENTATION_DN	49	Up	0.000122347	0.009041761	0.119858453	0	49	0.812407352
c2	EBV+ FK506+ vs. EBV– FK506+	ZHAN_MULTIPLE_MYELOMA_DN	24	Down	0.00020594	0.012625811	0.049529343	1	41	0.614570173
c7	EBV+ FK506+ vs. EBV– FK506+	GSE10325_LUPUS_BCELL_VS_LUPUS_MYELOID_DN	164	Down	1.13E-06	0.000263352	0.003229648	5	200	0.201018672
c7	EBV+ FK506+ vs. EBV– FK506+	GSE42724_NAIVE_BCELL_VS_PLASMABLAST_UP	161	Up	3.55E-05	0.003361542	0.00775475	9	199	0.309169645
c7	EBV+ FK506+ vs. EBV– FK506+	GSE22886_NAIVE_BCELL_VS_MONOCYTE_DN	183	Down	0.000238638	0.013949652	0.051543346	7	200	0.621587248
h	EBV+ FK506+ vs. EBV+ FK506–	HALLMARK_MYC_TARGETS_V2	58	Up	2.58E-14	7.83E-11	1.08E-08	4	58	1.63E-05
h	EBV+ FK506+ vs. EBV+ FK506–	HALLMARK_MYC_TARGETS_V1	199	Up	5.19E-09	2.24E-06	0.004456695	10	200	0.22674753
h	EBV+ FK506+ vs. EBV+ FK506–	HALLMARK_HEME_METABOLISM	166	Down	1.03E-05	0.001421078	0.123230012	10	200	0.821345159
h	EBV+ FK506+ vs. EBV+ FK506–	HALLMARK_E2F_TARGETS	193	Up	3.76E-05	0.003453056	0.469212981	7	200	0.980075513
h	EBV+ FK506+ vs. EBV+ FK506–	HALLMARK_HEDGEHOG_SIGNALING	17	Down	0.000629747	0.028911108	0.123820107	0	36	0.821345159
h	EBV+ FK506+ vs. EBV+ FK506–	HALLMARK_IL6_JAK_STAT3_SIGNALING	62	Down	0.001007466	0.037877129	0.004918596	1	87	0.237121162
c2	EBV+ FK506+ vs. EBV+ FK506–	SCHUHMACHER_MYC_TARGETS_UP	79	Up	8.42E-13	1.28E-09	7.87E-10	3	80	2.39E-06
c2	EBV+ FK506+ vs. EBV+ FK506–	DANG_MYC_TARGETS_UP	133	Up	9.52E-10	5.77E-07	2.70E-06	7	143	0.002047607
c2	EBV+ FK506+ vs. EBV+ FK506–	SCHLOSSER_MYC_TARGETS_AND_SERUM_RESPONSE_DN	46	Up	1.20E-09	6.04E-07	7.08E-07	3	47	0.000714707
c2	EBV+ FK506+ vs. EBV+ FK506–	PENG_LEUCINE_DEPRIVATION_DN	177	Up	2.49E-08	8.39E-06	0.001014079	6	187	0.128027494
c2	EBV+ FK506+ vs. EBV+ FK506–	PENG_GLUTAMINE_DEPRIVATION_DN	314	Up	2.78E-08	8.43E-06	0.005925323	9	337	0.267966084
c2	EBV+ FK506+ vs. EBV+ FK506–	HADDAD_B_LYMPHOCYTE_PROGENITOR	254	Down	1.22E-07	3.37E-05	0.003306601	6	293	0.201018672
c2	EBV+ FK506+ vs. EBV+ FK506–	BILD_MYC_ONCOGENIC_SIGNATURE	162	Up	1.23E-06	0.000265412	1.98E-05	8	206	0.008586439
c2	EBV+ FK506+ vs. EBV+ FK506–	PENG_RAPAMYCIN_RESPONSE_DN	231	Up	1.80E-06	0.000321177	0.013236694	13	245	0.387059286
c2	EBV+ FK506+ vs. EBV+ FK506–	PID_MYC_ACTIV_PATHWAY	73	Up	2.46E-06	0.000414326	0.00331714	5	79	0.201018672
c2	EBV+ FK506+ vs. EBV+ FK506–	ZHAN_MULTIPLE_MYELOMA_DN	24	Down	3.92E-05	0.003489062	0.002600576	1	41	0.201018672
c2	EBV+ FK506+ vs. EBV+ FK506–	TARTE_PLASMA_CELL_VS_PLASMABLAST_DN	298	Up	8.44E-05	0.006728949	0.02162392	16	309	0.488248953

c2	EBV+ FK506+ vs. EBV+ FK506–	ACOSTA_PROLIFERATION_INDEPENDENT_MYC_TARGETS_UP	76	Up	0.000144216	0.010323981	0.045714649	3	84	0.59704908
c2	EBV+ FK506+ vs. EBV+ FK506–	SCHLOSSER_MYC_TARGETS_AND_SERUM_RESPONSE_UP	47	Up	0.000172091	0.011487787	0.044383261	1	47	0.590320947
c2	EBV+ FK506+ vs. EBV+ FK506–	SCHLOSSER_MYC_TARGETS_REPRESSED_BY_SERUM	154	Up	0.000175218	0.011487787	0.117694716	11	159	0.811289041
c2	EBV+ FK506+ vs. EBV+ FK506–	HASLINGER_B_CLL_WITH_MUTATED_VH_GENES	12	Mixed	0.297252709	0.012613351	4.16E-05	0	18	0.012613351
c2	EBV+ FK506+ vs. EBV+ FK506–	GARY_CD5_TARGETS_DN	415	Up	0.0002394	0.013949652	0.591456579	13	431	0.999416688
c2	EBV+ FK506+ vs. EBV+ FK506–	TARTE_PLASMA_CELL_VS_B_LYMPHOCYTE_UP	72	Down	0.000669642	0.028985917	0.568409218	2	78	0.999416688
c2	EBV+ FK506+ vs. EBV+ FK506–	BASSO_B_LYMPHOCYTE_NETWORK	138	Up	0.000772472	0.031629604	0.003470659	5	143	0.202232608
c2	EBV+ FK506+ vs. EBV+ FK506–	BASSO_HAIRY_CELL_LEUKEMIA_DN	57	Down	0.000999303	0.037877129	0.037846937	3	80	0.581832186
c2	EBV+ FK506+ vs. EBV+ FK506–	DIRMEIER_LMP1_RESPONSE_LATE_UP	57	Up	0.001394247	0.047467047	0.078075984	2	57	0.712369661
c3	EBV+ FK506+ vs. EBV+ FK506–	TF_MYC_Q2	124	Up	0.001093033	0.040388913	0.05102674	9	185	0.621587248
c4	EBV+ FK506+ vs. EBV+ FK506–	MODULE_54	226	Up	0.000801597	0.032384504	0.757284249	5	263	0.999416688
c5	EBV+ FK506+ vs. EBV+ FK506–	GO_NUCLEOLAR_PART	59	Up	5.30E-10	4.02E-07	5.15E-05	5	62	0.014198037
c5	EBV+ FK506+ vs. EBV+ FK506–	GO_RAN_GTPASE_BINDING	27	Up	7.06E-05	0.005785034	0.004560866	0	31	0.22674753
c5	EBV+ FK506+ vs. EBV+ FK506–	GO_DEATH_RECEPTOR_ACTIVITY	17	Mixed	0.017373701	0.012613351	3.44E-05	0	24	0.012613351
c5	EBV+ FK506+ vs. EBV+ FK506–	GO_NUCLEOLUS	741	Up	0.000648975	0.028951521	0.152926304	39	848	0.88260324
c7	EBV+ FK506+ vs. EBV+ FK506–	GSE10325_LUPUS_BCELL_VS_LUPUS_MYELOID_DN	164	Down	5.92E-07	0.000149471	6.03E-05	5	200	0.015234936
c7	EBV+ FK506+ vs. EBV+ FK506–	GSE10325_BCELL_VS_MYELOID_DN	153	Down	4.16E-06	0.000641739	0.007503348	4	200	0.303135275
c7	EBV+ FK506+ vs. EBV+ FK506–	GSE3982_BCELL_VS_BASOPHIL_DN	132	Down	2.88E-05	0.003137071	0.074568802	4	200	0.703873736
c7	EBV+ FK506+ vs. EBV+ FK506–	GSE42724_NAIVE_VS_B1_BCELL_DN	167	Up	0.00010393	0.007872668	0.011091208	5	199	0.373404016
c7	EBV+ FK506+ vs. EBV+ FK506–	GSE12366_PLASMA_CELL_VS_NAIVE_BCELL_UP	142	Down	0.000467312	0.022475491	0.886310678	7	200	0.999416688
c7	EBV+ FK506+ vs. EBV+ FK506–	GSE12366_PLASMA_CELL_VS_MEMORY_BCELL_UP	137	Down	0.000724736	0.030928893	0.898162131	5	200	0.999416688
c7	EBV+ FK506+ vs. EBV+ FK506–	GSE22886_NAIVE_BCELL_VS_BM_PLASMA_CELL_DN	162	Down	0.000772126	0.031629604	0.447382286	4	200	0.980075513
c7	EBV+ FK506+ vs. EBV+ FK506–	GSE3982_EOSINOPHIL_VS_BCELL_UP	118	Down	0.001012557	0.037877129	0.119431822	4	200	0.812407352
c7	EBV+ FK506+ vs. EBV+ FK506–	GSE22886_NAIVE_BCELL_VS_MONOCYTE_DN	183	Down	0.001152519	0.041839955	0.018238915	7	200	0.445676714
c7	EBV+ FK506+ vs. EBV+ FK506–	GSE30153_LUPUS_VS_HEALTHY_DONOR_BCELL_DN	110	Down	0.001213439	0.042752568	0.077630905	3	200	0.712369661

c7	EBV+ FK506+ vs. EBV+ FK506–	GSE22886_IGG_IGA_MEMORY_BCELL_VS_BM_PLASMA_CELL_DN	152	Down	0.001357414	0.046738237	0.187173846	7	200	0.892294232
c7	EBV+ FK506+ vs. EBV+ FK506–	GSE22886_IGM_MEMORY_BCELL_VS_BM_PLASMA_CELL_DN	157	Down	0.001425334	0.047986259	0.253725815	9	200	0.934838348
c7	EBV+ FK506+ vs. EBV+ FK506–	GSE10325_LUPUS_CD4_TCELL_VS_LUPUS_BCELL_UP	172	Down	0.001478154	0.049217651	0.095154273	10	200	0.763051295
h	EBV+ FK506+ vs. (EBV+ FK506– & EBV– FK506+)	HALLMARK_INTERFERON_ALPHA_RESPONSE	92	Up	4.24E-06	0.000641739	0.040228219	7	97	0.581832186
h	EBV+ FK506+ vs. (EBV+ FK506– & EBV– FK506+)	HALLMARK_MYC_TARGETS_V2	58	Up	0.000279983	0.014883322	0.014510032	4	58	0.389074308
h	EBV+ FK506+ vs. (EBV+ FK506– & EBV– FK506+)	HALLMARK_CHOLESTEROL_HOMEOSTASIS	58	Up	0.000840721	0.033518223	0.001140183	1	74	0.138190231
c2	EBV+ FK506+ vs. (EBV+ FK506– & EBV– FK506+)	TARTE_PLASMA_CELL_VS_B_LYMPHOCYTE_UP	72	Down	2.91E-05	0.003137071	0.331798512	2	78	0.959158425
c2	EBV+ FK506+ vs. (EBV+ FK506– & EBV– FK506+)	PID_MYC_ACTIV_PATHWAY	73	Up	0.000177761	0.011487787	0.110605786	5	79	0.807396648
c2	EBV+ FK506+ vs. (EBV+ FK506– & EBV– FK506+)	ZHAN_MULTIPLE_MYELOMA_DN	24	Down	0.000207827	0.012625811	0.007990753	1	41	0.310410015
c2	EBV+ FK506+ vs. (EBV+ FK506– & EBV– FK506+)	SCHUHMACHER_MYC_TARGETS_UP	79	Up	0.000208347	0.012625811	0.053316921	3	80	0.621587248
c2	EBV+ FK506+ vs. (EBV+ FK506– & EBV– FK506+)	PENG_LEUCINE_DEPRIVATION_DN	177	Up	0.000325805	0.01702048	0.049648191	6	187	0.614570173
c5	EBV+ FK506+ vs. (EBV+ FK506– & EBV– FK506+)	GO_DEATH_RECEPTOR_ACTIVITY	17	Up	0.000257058	0.014198926	0.002086315	0	24	0.176169164
c5	EBV+ FK506+ vs. (EBV+ FK506– & EBV– FK506+)	GO_NEGATIVE_REGULATION_OF_ENDOPLASMIC_RETICULUM_STRESS_INDUCED_INTRINSIC_APOPTOTIC_SIGNALING_PATHWAY	15	Down	0.000446969	0.02220188	0.22831325	0	19	0.92692861
c5	EBV+ FK506+ vs. (EBV+ FK506– & EBV– FK506+)	GO_REGULATION_OF_ENDOPLASMIC_RETICULUM_STRESS_INDUCED_INTRINSIC_APOPTOTIC_SIGNALING_PATHWAY	25	Down	0.000459821	0.022471891	0.614485717	0	30	0.999416688
c5	EBV+ FK506+ vs. (EBV+ FK506– & EBV– FK506+)	GO_ISOTYPE_SWITCHING	15	Up	0.001245433	0.043375435	0.108872295	0	16	0.802430644
c7	EBV+ FK506+ vs. (EBV+ FK506– & EBV– FK506+)	GSE42724_NAIVE_BCELL_VS_PLASMABLAST_UP	161	Up	1.49E-06	0.000282732	0.000997395	9	199	0.128027494
c7	EBV+ FK506+ vs. (EBV+ FK506– & EBV– FK506+)	GSE12366_PLASMA_CELL_VS_NAIVE_BCELL_UP	142	Down	6.03E-06	0.000870446	0.087604297	7	200	0.747600301
c7	EBV+ FK506+ vs. (EBV+ FK506– & EBV– FK506+)	GSE22886_IGM_MEMORY_BCELL_VS_BM_PLASMA_CELL_DN	157	Down	1.55E-05	0.001946971	0.06802993	9	200	0.664937704
c7	EBV+ FK506+ vs. (EBV+ FK506– & EBV– FK506+)	GSE22886_NAIVE_BCELL_VS_BM_PLASMA_CELL_DN	162	Down	1.61E-05	0.001946971	0.166203947	4	200	0.888949924
c7	EBV+ FK506+ vs. (EBV+ FK506– & EBV– FK506+)	GSE12366_PLASMA_CELL_VS_MEMORY_BCELL_UP	137	Down	3.29E-05	0.003217248	0.341254563	5	200	0.959158425
c7	EBV+ FK506+ vs. (EBV+ FK506– & EBV– FK506+)	GSE10325_LUPUS_BCELL_VS_LUPUS_MYELOID_DN	164	Down	4.23E-05	0.003657695	0.001481886	5	200	0.149670531
c7	EBV+ FK506+ vs. (EBV+ FK506– & EBV– FK506+)	GSE22886_IGG_IGA_MEMORY_BCELL_VS_BM_PLASMA_CELL_DN	152	Down	0.000262422	0.014198926	0.082569384	7	200	0.733681038
c7	EBV+ FK506+ vs. (EBV+ FK506– & EBV– FK506+)	GSE3982_BCELL_VS_TH2_DN	133	Up	0.000560133	0.026110811	0.115362516	6	200	0.811289041
c7	EBV+ FK506+ vs. (EBV+ FK506– & EBV– FK506+)	GSE22886_NAIVE_BCELL_VS_MONOCYTE_DN	183	Down	0.000659292	0.028951521	0.012190037	7	200	0.387059286
