# Supplementary material for: Immunosuppressive FK506 treatment leads to more frequent EBV-associated lymphoproliferative disease in humanized mice
Source: PLoS Pathog. 2020 Apr 6;16(4):e1008477. doi: 10.1371/journal.ppat.1008477 (PMC7162544; doi:10.1371/journal.ppat.1008477)
Supplement: S3 Table — (PDF) [file ppat.1008477.s004.pdf]

S3 Table. PTLD patients' characteristics.

Summary	Gender	Number of subjects	Age (years)	
			Mean ± SD	Median [Range]
	F	7	2.29 ± 0.95	2.42 [1.21 – 3.51]
	M	6	4.41 ± 4.31	3.05 [0.75 – 12.6]
**Total**	F:M ratio **1.17**	**13**	**3.27 ± 3.07**	**2.42 [0.75 – 12.6]**

Details

No.	Type of Transplant	Type of PTLD	Acute Time-Point*	Recovery Time-Point*	Analysis
**1**	Liver	IM-like PTLD	0m	48m	*a, b*
**2**	Liver	IM-like PTLD	0.5m	36m	*a, b*
**3**	Liver	PTLD, plasmacytic hyperplasia	0m	36m	*a, b*
**4**	Liver	Monomorphic PTLD	0.25m	48m	*a, b*
**5**	Liver	IM-like PTLD	0.25m	45m	*a, b*
**6**	Liver	IM-like PTLD	0m	42m	*a, b*
**7**	Liver	Monomorphic PTLD (Burkitt lymphoma)	0m	45m	*a, b*
**8**	Liver and stem cells	Atypical PTLD; lymphoid infiltrate with a majority of $EBER_+$ $CD3_+$ $CD8_+$ cells	0m	45m	*a, b*
**9**	Liver	IM-like PTLD	0.25m	48m	*a*
**10**	Liver	IM-like PTLD	0m	36m	*a*
**11**	Liver	IM-like PTLD	1m	36m	*a*
**12**	Liver	IM-like PTLD	3m	42m	*a*
**13**	Liver	IM-like PTLD	2m	42m	*a*

***** months post diagnosis
a sCD30 serum concentration
b PRR4 and cytokine serum concentration
