# Supplementary material for: Immunosuppressive FK506 treatment leads to more frequent EBV-associated lymphoproliferative disease in humanized mice
Source: PLoS Pathog. 2020 Apr 6;16(4):e1008477. doi: 10.1371/journal.ppat.1008477 (PMC7162544; doi:10.1371/journal.ppat.1008477)
Supplement: S4 Table — (PDF) [file ppat.1008477.s005.pdf]

S4 Table. Control subjects' characteristics.

Cohort	Gender	Number of subjects	Age (years)		% EBV VCA-IgG seropositive
			Mean ± SD	Median [Range]	
**Tonsillectomy**	F:M ratio **1.07**	**31**	**3.92 ± 2.96**	**3.12 [0.94 – 12.85]**	**51.6%**
	F	16	3.19 ± 0.68	3.19 [2.07 – 4.51]	50%
	M	15	4.71 ± 4.13	3.04 [0.94 – 12.85]	53.3%
**Febrile non-EBV**	F:M ratio **1.33**	**21**	**3.15 ± 2.51**	**2.64 [0.33 – 12.67]**	**52.4%***
	F	12	2.44 ± 0.86	2.38 [1 – 3.75]	50%
	M	9	4.11 ± 3.59	3.00 [0.33 – 12.67]	55.6%
Total	F:M ratio **1.17**	**52**	**3.61 ± 2.79**	**2.99 [0.33 – 12.85]**	**51.9%**

***** all subjects were tested negative for EBV VCA-IgM
