# Supplementary figures and images for: Immunosuppressive FK506 treatment leads to more frequent EBV-associated lymphoproliferative disease in humanized mice
Source: PLoS Pathog. 2020 Apr 6;16(4):e1008477. doi: 10.1371/journal.ppat.1008477 (PMC7162544; doi:10.1371/journal.ppat.1008477)

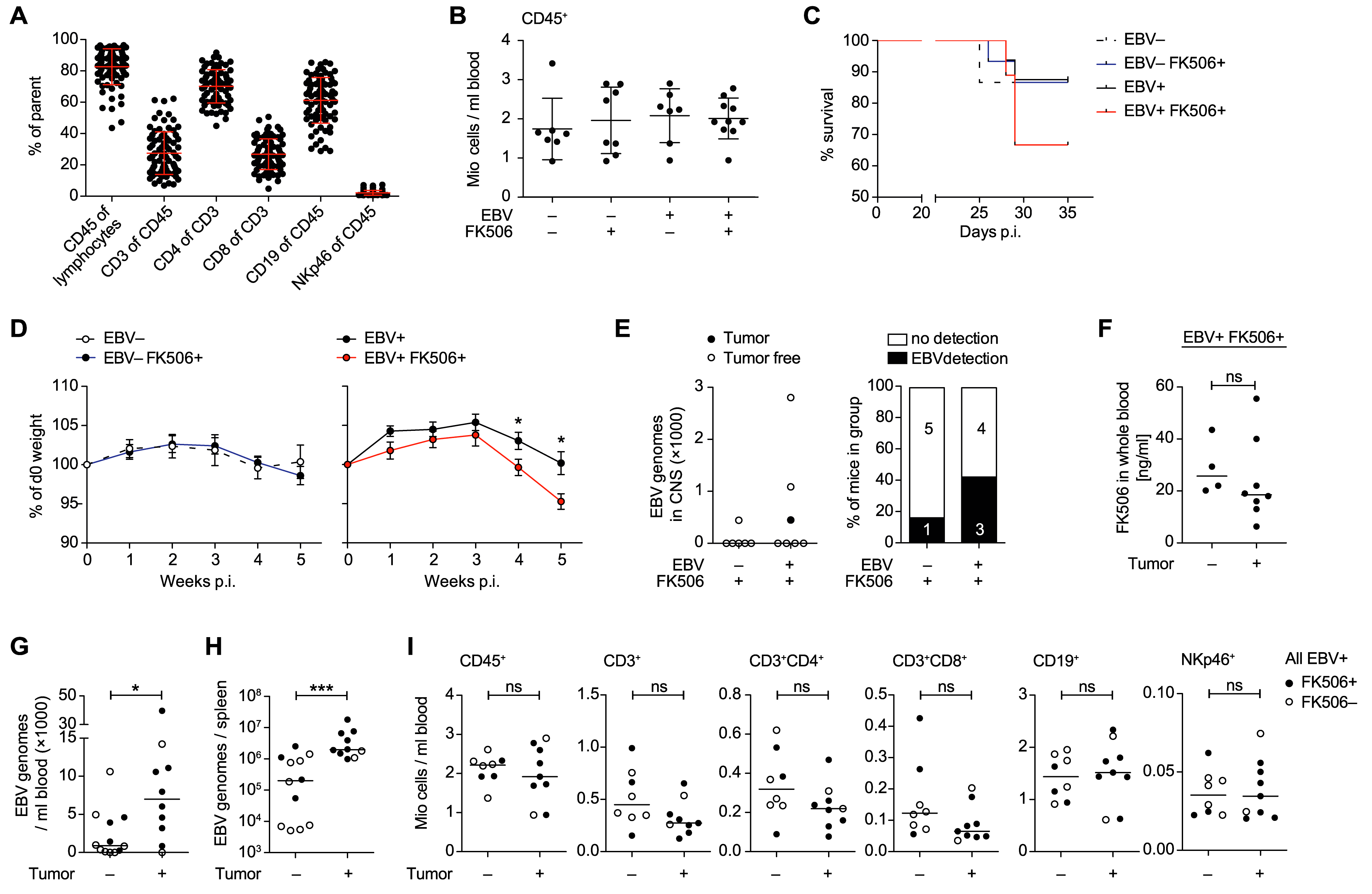

Supplement: S1 Fig — A) Baseline peripheral blood human lymphocyte engraftment in huNSG-A2 mice before infection. Composite data of five independent experiments (n = 80) each reconstituted with CD34+ cells from a single HFL donor (n = 5). Percentages given represent values of human CD45+ lymphocytes; CD3+, CD19+ and CD3−NKp46+ cells within the CD45+ population, and percentages of CD4+ and CD8+ cells of CD3+ T cells. B) Total numbers of human CD45+ cells per ml blood before infection is depicted for individual mice (n = 7–10 animals per group). C) Composite survival from four independent experiments is depicted for the indicated groups. Mice were euthanized when weight loss exceeded 15% of the maximum weight or when signs of morbidity necessitated a premature euthanasia based on the laboratory’s animal welfare protocol (n = 15–18 animals per group). D) Relative weight development of mice that survived until five weeks p.i. represented as mean ± SEM percent of starting weight per group for four pooled experiments; EBV−FK506– vs. EBV−FK506+ p = 0.6474, p = 0.4496 for week four and five respectively; EBV+ FK506– vs. EBV+ FK506+ p = 0.0354 and p = 0.0128 for week four and five respectively (unpaired t tests). E) Analysis of EBV BamHI W fragment DNA detection as determined by qPCR in lymphocytes derived from the CNS after PBS perfusion at five weeks p.i.. Composite data from two independent experiments with EBV+ FK506– (n = 6) and EBV+ FK506+ (n = 7) depicted as raw viral titers and percent of mice per group with DNA levels above the qPCR detection threshold. F) Blood FK506 levels (ng/ml) in EBV-infected mice with (n = 8) and without (n = 4) macroscopically visible tumors. Median, MWT p = 0.2303. G) EBV BamHI W DNA burden in the blood and H) in the spleen, measured at the day of sacrifice, in infected mice with (n = 10) and without (n = 12) macroscopically visible tumors. Median, MWT p = 0.0298 and p = 0.0004, respectively. F-H) Composite data from three independent experiments in which mice [file ppat.1008477.s006.tif]

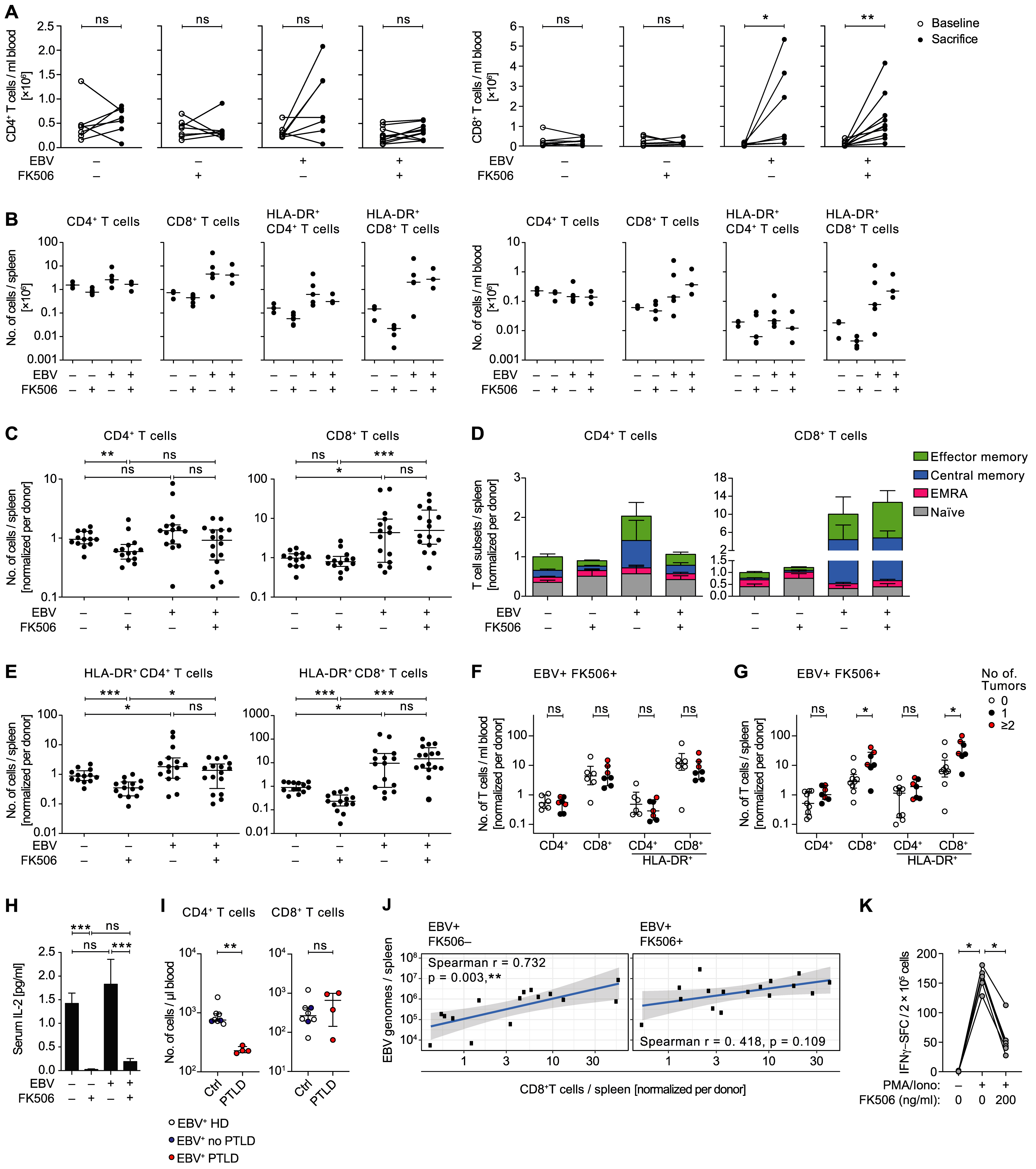

Supplement: S2 Fig — A) The numbers of total CD4+ or CD8+ T cells in the blood at baseline and 5 weeks p.i. are depicted for individual mice of two independent experiments (n = 7–10 animals per group), Wilcoxon signed-rank test. B) Total numbers of T cell subsets in the spleen (left) and blood (right) in mice from a representative experiment with n = 3–6 animals per groups. Median. C) The number of total CD4+ and CD8+ T cells in the spleen is plotted relative to the mean total CD4+ or CD8+ T cells of the EBV−FK506– group of each HPC donor. Median (IQR), MWT. D) Differentiation status of T cells in the spleen is represented as stacked bar graphs (n = 7–10 animals per group). Naïve: CD62L+ CD45RA+, central memory: CD62L+ CD45RA−, effector memory: CD62L− CD45RA−, effector memory RA+ (EMRA): CD62L− CD45RA+. E) Relative counts of activated CD4+ or CD8+ T cells in the spleen were determined by HLA-DR+ surface staining. Median (IQR), MWT. F-G) Relative counts of total and activated (HLA-DR+) CD4+ and CD8+ T cells are depicted for F) the blood and G) the spleen of tumor-bearing and tumor-free mice of the EBV+ FK506+ group from three independent experiments. Cell numbers are presented relative to the respective mean cell count of the EBV−FK506– group of each HPC donor. Tumor presence in individual mice is indicated by symbol color: clear = no tumors, black = 1 tumor, red = 2 or more tumors. Median (IQR), MWT. H) IL-2 concentration (pg/ml) was measured in the serum obtained on the day of sacrifice. EBV−FK506+ (n = 5), EBV−FK506+ (n = 8), EBV+ FK506– (n = 6), EBV+ FK506+ (n = 10). Median (IQR), MWT. I) Blood CD4+ and CD8+ T cell counts in PTLD patients and controls (Ctrl) derived from data previously reported by Sebelin-Wulf et al. [26]. Healthy EBV positive controls (EBV+ HD, n = 6), EBV positive transplant recipients without PTLD development (EBV+ no PTLD, n = 2), PTLD patients with histologically verified EBV association (EBV+ PTLD, n = 4). Median (IQR), MWT. J) Correlations between the relativ [file ppat.1008477.s007.tif]

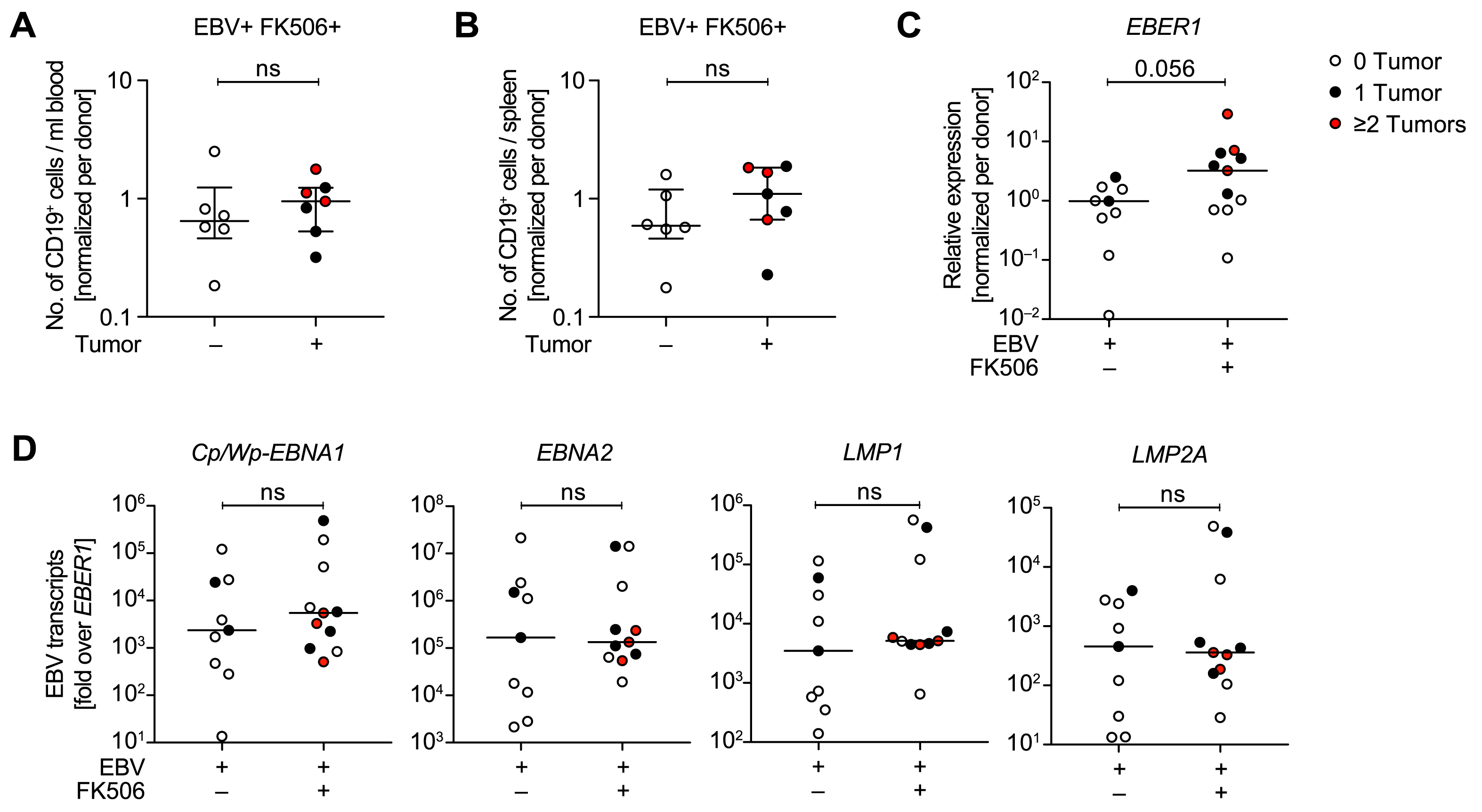

Supplement: S3 Fig — The number of CD19+ cells in A) blood and B) spleen is depicted for tumor-bearing and tumor-free mice of the EBV+ FK506+ group from three independent experiments. CD19+ cell counts are presented relative to the mean CD19+ cell count of the EBV−FK506– group of each HPC donor. Median (IQR), MWT. C-D) EBV transcript expression as measured by RT-qPCR in CD19+ splenocytes is depicted for individual mice from three independent experiments with EBV+ FK506– (n = 9) and EBV+ FK506+ (n = 11). C) EBER1 expression relative to the 18S rRNA reference gene and normalized per donor. D) Cp/Wp-EBNA1, EBNA2 and LMP1 and -2A transcript expression normalized to EBER1. Median, MWT. A-D) Tumor presence in individual mice is indicated by symbol color: clear = no tumors, black = 1 tumor, red = 2 or more tumors. (TIF) [file ppat.1008477.s008.tif]

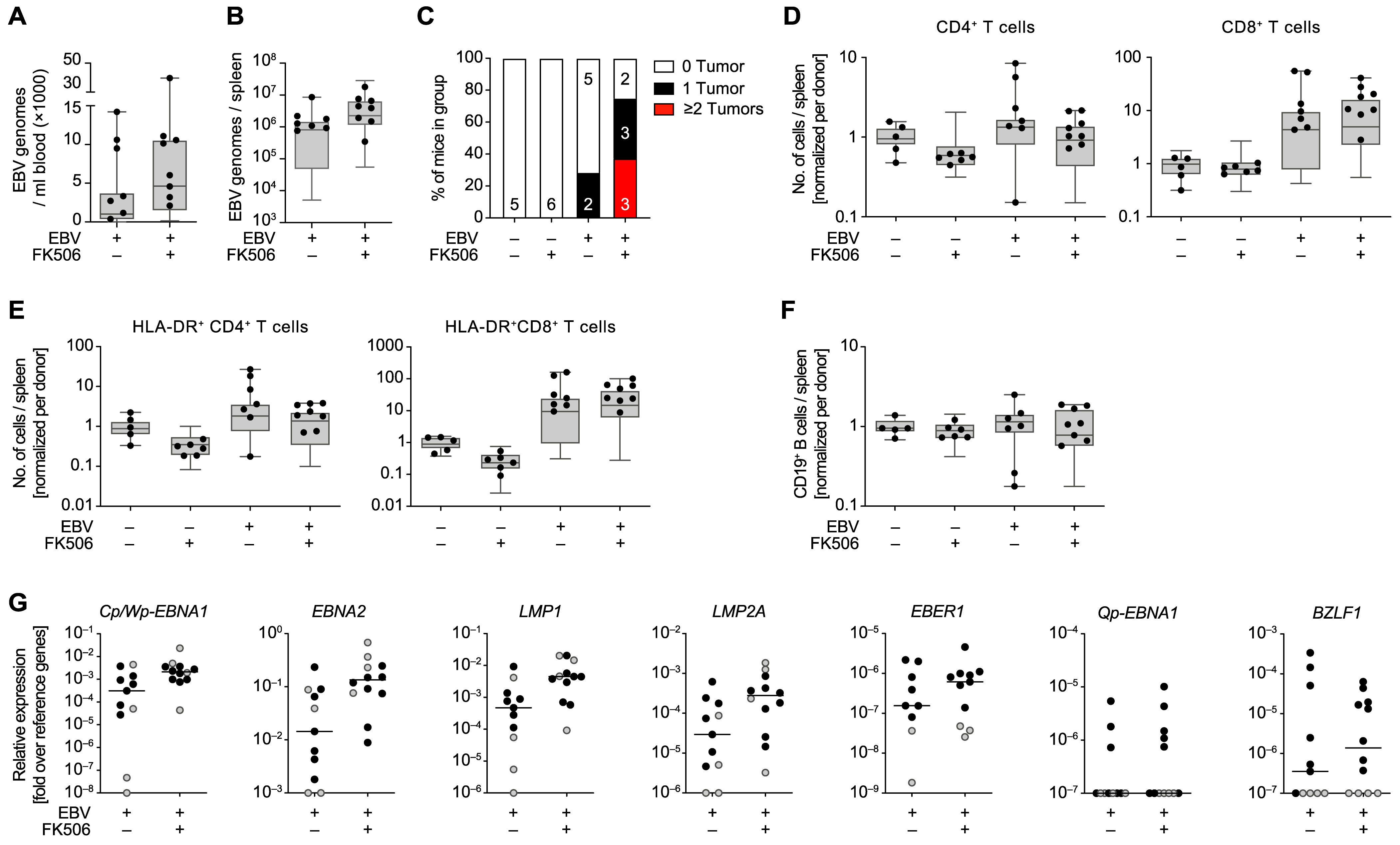

Supplement: S4 Fig — A) EBV DNA load in whole blood and B) in the spleen of mice at the day of sacrifice. C) Percent of all mice included in the AmpliSeq RNA profiling with macroscopically visible tumors with absolute numbers of mice indicated per group. D) Total numbers T cells and E) activated T cells per spleen normalized per donor. F) Total splenic CD19+ cell counts normalized per donor. A-B, D-F) Black dots indicate individual mice included in the RNA profiling analysis. Grey boxplots indicate median (IQR) and range of the total respective data set presented in Figs 1, 3 and S2. G) EBV transcript expression relative to the geometric mean of GAPDH and SDHA levels. EBER1 expression relative to the 18S rRNA reference gene. Mice included in the RNA profiling analysis are depicted as black symbols; other mice of the total respective data set presented in Figs 3 and S3 are depicted as grey symbols. (TIF) [file ppat.1008477.s009.tif]

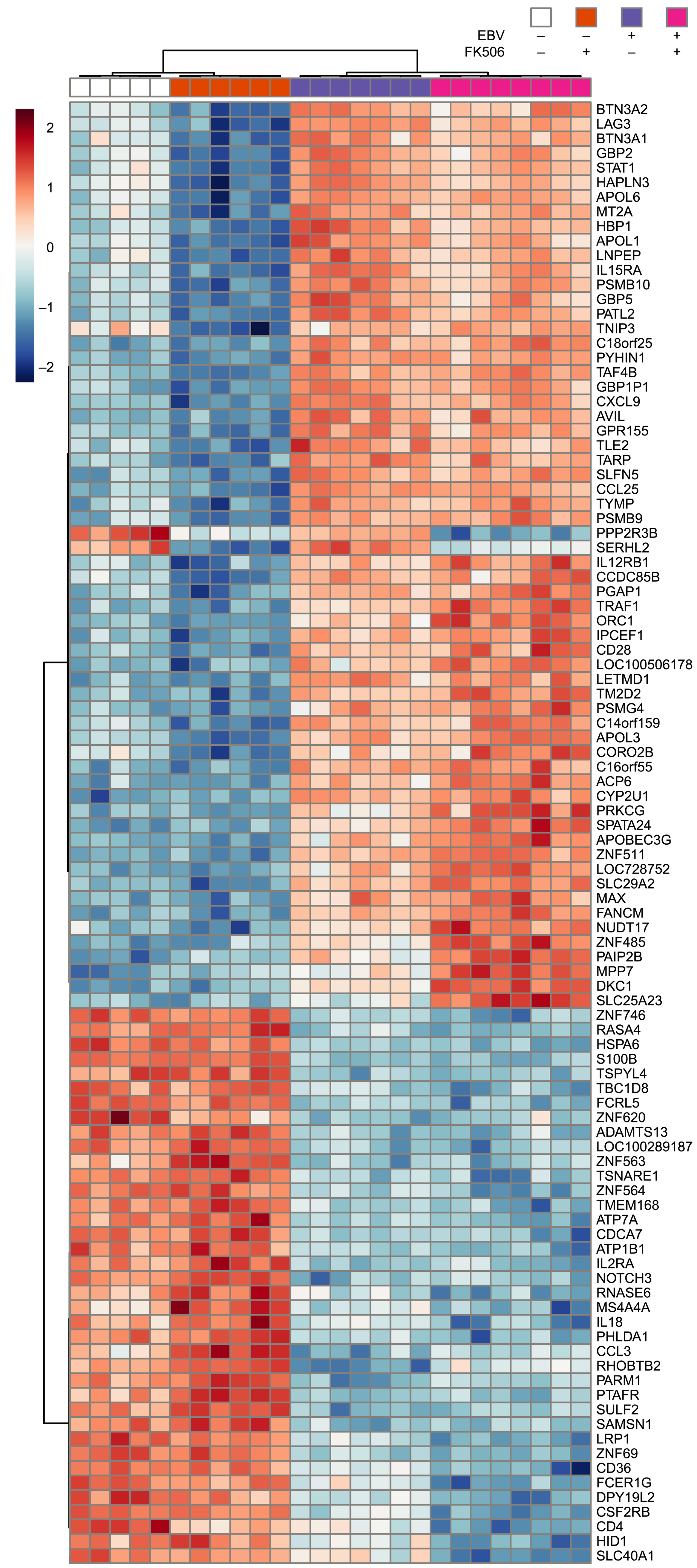

Supplement: S5 Fig — Heatmap of the gene expression vs. sample matrix from RNA expression profiling of the human B cell transcriptome of mice from three independent experiments with EBV−FK506– (n = 5, clear), EBV−FK506+ (n = 6, orange), EBV+ FK506– (n = 7, purple) and EBV+ FK506+ (n = 8, magenta). Displayed are the top 100 differentially expressed genes (DEGs) of all samples (i.e. individual mice). Hierarchical clustering separates genes with positive log fold change from negative and tries to group different sample types. (TIF) [file ppat.1008477.s010.tif]

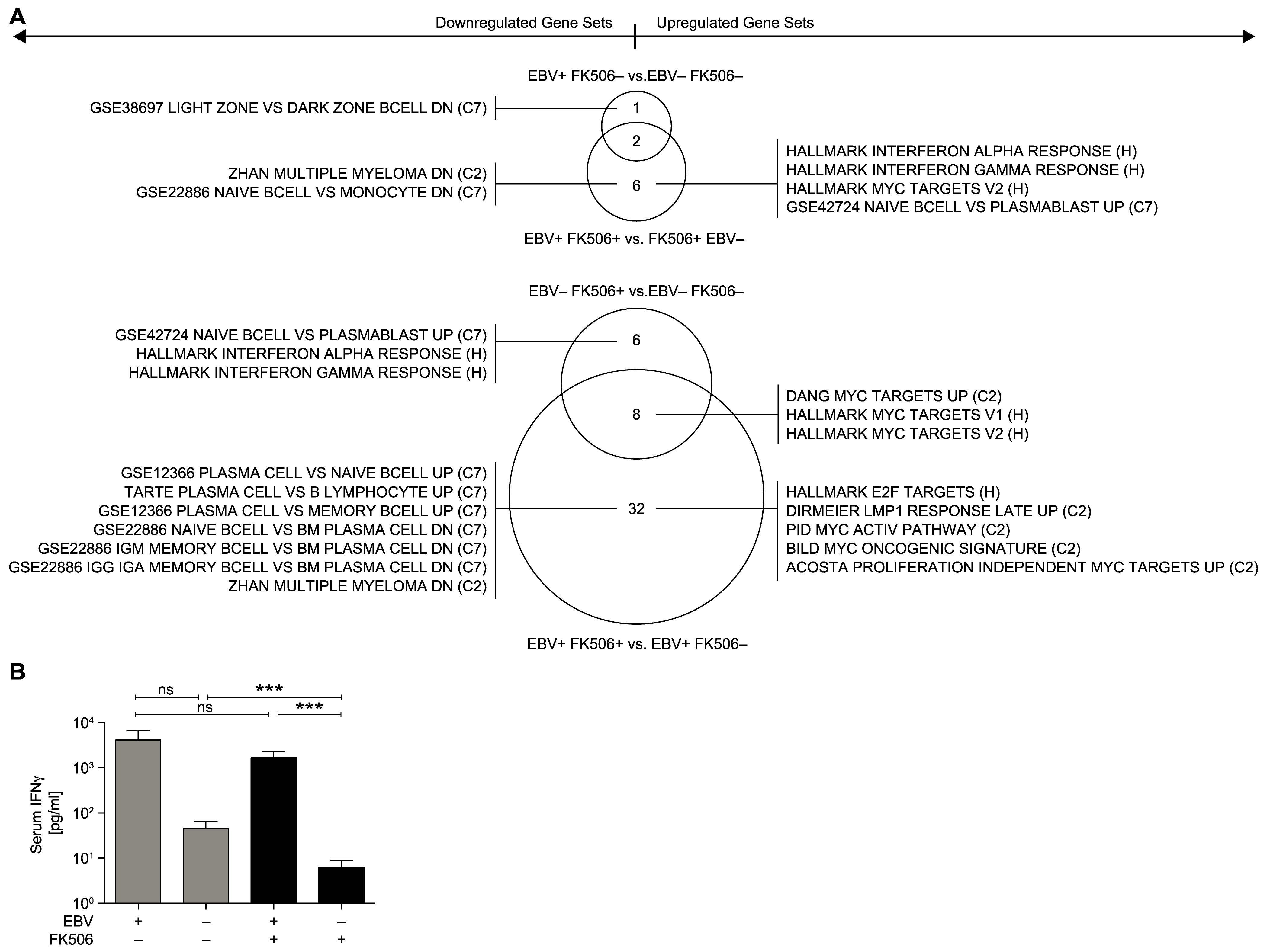

Supplement: S6 Fig — A) Venn diagrams depicting the number of enriched gene sets for the four comparisons EBV+ FK506– vs. EBV−FK506– and EBV+ FK506+ vs. EBV−FK506+ (upper panel), and EBV−FK506+ vs. EBV−FK506– and EBV+ FK506+ vs. EBV+ FK506– (lower panel). Selected up-regulated gene sets are indicated to the right, down-regulated gene sets to the left. B) IFNγ concentration (pg/ml) was measured in the serum obtained on the day of sacrifice. EBV−FK506– (n = 5), EBV−FK506+ (n = 8), EBV+ FK506– (n = 6), EBV+ FK506+ (n = 10). Mean ± SEM, MWT. ***: p<0.001. (TIF) [file ppat.1008477.s011.tif]

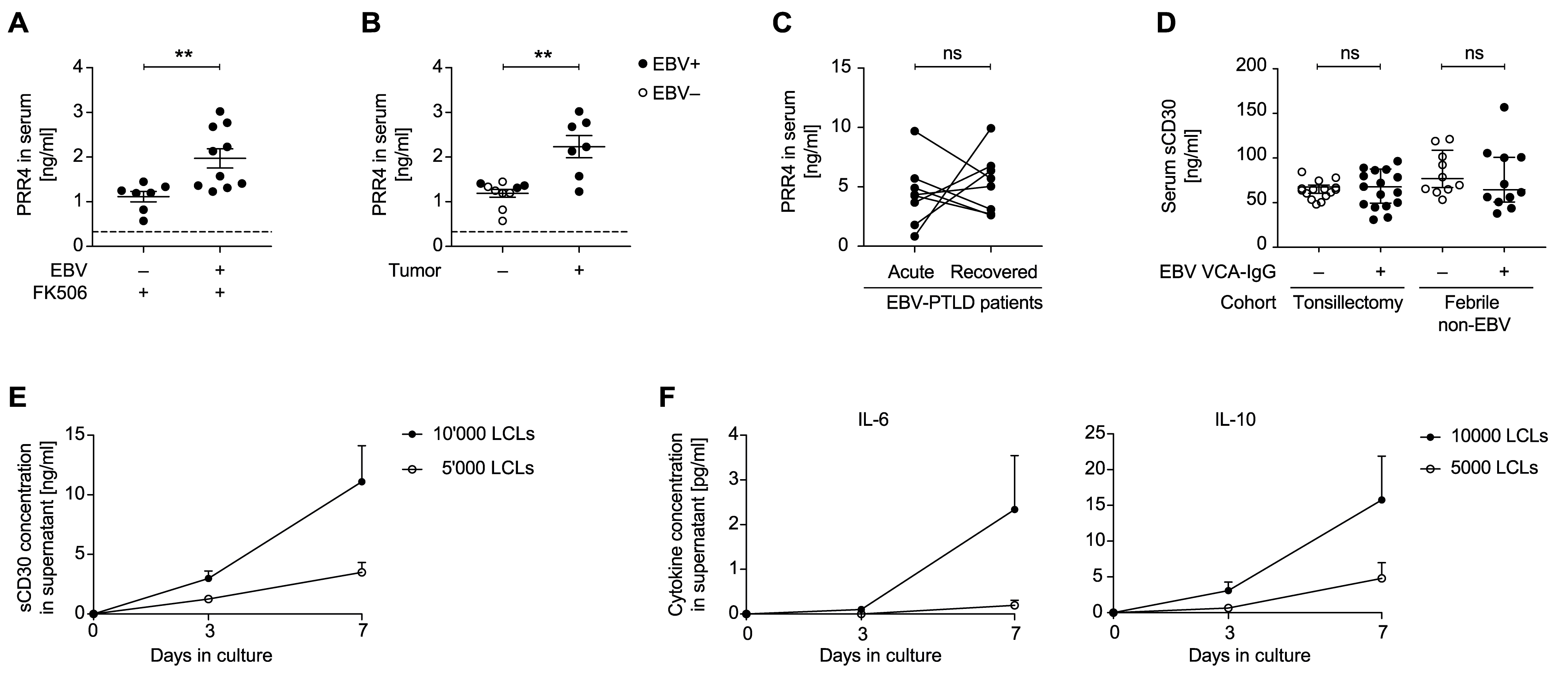

Supplement: S7 Fig — A-C) Protein concentration of PRR4 was measured in serum samples with ELISA. A) Serum was obtained at the day of sacrifice from animals of the indicated groups and are depicted from two independent experiments. Dashed lines indicate quantification thresholds. Mean ± SEM, MWT. B) Post hoc stratification of FK506-treated mice with or without macroscopically visible tumors with EBV-infection status indicated by black (EBV+) or clear (EBV–) symbols. Mean ± SEM, MWT. C) PRR4 was measured in the sera of 8 pediatric PTLD patients at diagnosis and again upon recovery at least 36 months later (see S3 Table). Wilcoxon matched-pairs test. D) sCD30 was measured in the sera of 31 healthy children undergoing elective tonsillectomy and 21 children that presented with non-EBV associated fever (Febrile non-EBV, EBV VCA-IgM negative). Approximately 50% of each of these control cohorts were positive for EBV VCA-IgG (see S4 Table). Median (IQR), MWT. E) Concentrations of sCD30 and F) IL-6 and IL-10 were measured in the supernatant of LCLs obtained by the ex vivo expansion of cells from individual EBV-infected humanized mice (n = 4). LCLs were seeded at 5000 or 10000 cells and supernatant was collected after 0, 3 and 7 days of culture. **: p<0.01. (TIF) [file ppat.1008477.s012.tif]
