# Supplementary material for: Immunosuppressive FK506 treatment leads to more frequent EBV-associated lymphoproliferative disease in humanized mice
Source: PLoS Pathog. 2020 Apr 6;16(4):e1008477. doi: 10.1371/journal.ppat.1008477 (PMC7162544; doi:10.1371/journal.ppat.1008477)
Supplement: Attachment — Submitted filename: point-by-point 131219.docx [file ppat.1008477.s013.docx]

Point by point response

*Reviewer remarks in Black italics.*

Author’s response in Green

We thank all three reviewers for their supportive and constructive comments, which we have integrated into the revised manuscript. Please find the detailed answers below.

**Part I – Summary**

*Reviewer #1: This manuscript uses a humanized mouse model to examine whether FK506 treatment (a calcineurin inhibitor often used to immunosuppress transplant patients) increases the ability of EBV infection to induce lymphomas in vivo. It has been previously shown by other groups that various types of agents that inhibit T cell function increase the ability of EBV to induce lymphomas in humanized mice, since T cells have some ability to control EBV in these models. Not surprisingly, the authors find that EBV-infected mice treated with FK506 (a known T cell function inhibitor) develop more frequent tumors and have higher viral loads. RNAseq analysis in EBV-infected animals treated with and without FK506 identifies CD30 (a cellular gene already well known to be stimulated by EBV infection of B cells, and already shown to be increased in the plasma of patients with mononucleosis and PTLD) as being increased in the EBV-infected mice treated with FK506. The authors then confirm that levels of soluble CD30 may be a good biomarker for detecting EBV+ PTLD in transplant patients. Over-all the study is well performed and the data are of high quality. However, the results are not particularly novel or surprising and provide only incremental advances to previously published literature.*

We thank Reviewer #1 for reading our manuscript in detail and assessing it in the context of previously published studies. We also welcome the remarks on the data quality and overall performance of the study. We mostly agree with Reviewer #1’s assessment of our study. However, we would like to point out that while the results may not be particularly novel in terms of the specific biomarker we validated in pediatric liver transplant patients at diagnosis and upon recovery (sCD30), we came to these results via a new disease model with a bottom-up approach. We find the identification of sCD30 as our top hit rather confirms that certain aspects of human PTLD can be modeled in huNSG mice. To date most studies inhibiting T cells in humanized mice have used monoclonal Antibodies to outright deplete total T cells or CD4^+^ and CD8^+^ cells individually or block receptors on their surface (some of these studies were performed by our group e.g. Strowig et al., J Exp Med 2009 and Chijioke et al., J Infect Dis 2015). In contrast, the modification of T cell function via small molecule inhibitors during EBV infection has not been documented in humanized mice. In anticipation of further studies using novel small molecule inhibitors in humanized mice, our study using the well-known calcineurin inhibitor FK506, will be a useful resource for effect size estimation, cohort size calculations and provides a B cell transcriptome dataset that can be used in comparison. We also probe and highlight the limits of this model by investigating other candidate biomarkers identified via Ampliseq expression profiling (see PRR4). We hope Reviewer 1 can see the value of an animal model that is not surprising, but all the same provides advances in our understanting of PTLDs and a platform to assess PTLD risk upon immune regulation.

*Reviewer #2: In this interesting manuscript the authors develop a model of Epstein Barr virus associated post-transplant lymphoproliferative disease (EBV-LPD) in humanized mice. They show, using state of the art technologies, the effect of tacrolimus (FK506)-mediated immunosuppression. While the current conclusions are not particular novel, the model represents a significant advance since it will allow for the first time the proper preclinical modeling of this complication post hematopoietic and solid organ transplant. Thus, it needs to be considered a significant advance in the field. The authors studies are comprehensive and are well designed. Data sets are analyzed meticulously and are experiments have appropriate controls. The conclusions of the authors are solid and are supported by the presented data. It is one of the few manuscripts I have reviewed within the last 5 years for which I have no concerns.*

We thank Reviewer #2 for the positive evaluation of our manuscript. We also hope that this model or variations thereof can be used to spur further advances in the fields of EBV-, LPD- and transplant immunology.

*Reviewer #3: This article presents a large variety of interesting and detailed observations and data regarding the biological consequences of experimental EBV infection in a humanized mouse model after application of the calcineurin inhibitor FK506. Since only a minority of animals develops tumors, it may be that this model is not predominantly a model of PTLD, depending on how that term is defined. Nontheless, the model has many interesting features. Since only some animals had tumors, it would be interesting to learn more about the correlates and parameters associated with tumor formation, based on the data already available to the authors. The authors have analyzed a multitude of parameters regarding immune cell reconstitution and activation, viral titers, viral and cellular gene expression etc. Not only tumor formation but also some other of these parameters vary quite widely within a treatment group, especially the EBV+FK506 group. It would be nice to learn more about how these parameters co-varied within a group. Maybe the authors can find ways to present their data that make such correlations more transparent.*

We thank Reviewer #3 for the detailed evaluation of our manuscript and the interest shown in our observations and data. We believe that the modifications made to the manuscript based on many of the Reviewer’s suggestions and questions has enhanced the clarity of the manuscript overall and gives the reader more descriptive information on how certain parameters co-varied based on tumor presence. We have addressed the Reviewer’s individual remarks in a point-by-point fashion below. We hope the reviewer can appreciate that we have undertaken every effort to address each concern or question.

**Part II – Major Issues: Key Experiments Required for Acceptance**

Please use this section to detail the key new experiments or modifications of existing experiments that should be absolutely required to validate study conclusions.

Generally, there should be no more than 3 such required experiments or major modifications for a "Major Revision" recommendation. If more than 3 experiments are necessary to validate the study conclusions, then you are encouraged to recommend "Reject".

*Reviewer #1: (No Response)*

*Reviewer #2: As outlined in the summary, believe that the developed model is a great step forward in the preclinical modeling of EBV-LPD. I have no major or minor concerns with the submitted manuscript.*

*Reviewer #3: (No Response)*

**Part III – Minor Issues: Editorial and Data Presentation Modifications**

Please use this section for editorial suggestions as well as relatively minor modifications of existing data that would enhance clarity.

*Reviewer #1: (No Response)*

*Reviewer #2: None*

*Reviewer #3: Individual remarks*

*title, "exacerbates" - this title does not seem to fit the observations very well. The authors found that FK506 leads to "elevated viral burden, more frequent tumor formation and diminished EBV-induced T cell responses" - but this is not the same as saying that PTLD was exacerbated, as opposed to raised in incidence. For example, it is not documented whether tumors were larger in FK506-treated animals. Weight loss was more pronounced; virus titers were higher; but B cell number was not increased.*

We agree with the Reviewer’s reasoning on the word “exacerbates” and have now modified the title to more accurately reflect the observations we made in this study. It now reads: Immunosuppressive FK506 treatment leads to more frequent EBV-associated lymphoproliferative disease in humanized mice.

Based on the Reviewer’s suggestion regarding the title, we have also modified the concluding sentence of the first results paragraph: Taken together, this indicates that FK506 treatment of EBV-infected huNSG-A2 mice leads to more frequent EBV-induced disease.

*p71, "with" missing*

This missing word has been corrected. It now reads as follows: Expression of EBNA1, LMP1 and LMP2 (latency II) rescues differentiating germinal center B cells allowing them to gain access to the memory B cell pool, where EBV persists without the expression of viral proteins (latency 0) or with transient EBNA1 expression (latency I) during homeostatic proliferation [6-8].

*Fig. 1B, does it show FK506 levels 24h after the *first* administration of FK506?*

FK506 measurements from whole blood were performed after a minimum of three applications and 24h after the last administration. The Figure caption has now been modified to include this information, it reads as follows: FK506 concentration was measured 24h after subcutaneous (s.c) administration of the compound mixture and after a minimum of three applications as described in A).

The method section has been modified to include this information, it now reads: FK506 concentration was measured after a minimum of three applications (i.e. one week of treatment) and 24h after the last subcutaneous administration.

*Fig. S1A shows only percentages. How good was human cell reconstitution really, i.e., what were absolute numbers of hCD45+ cells?*

We have now included the absolute number of human CD45^+^ cells in the blood for the different groups at the beginning of the experiment from all mice for which this data was available in the new Figure S1B. We also provide corresponding absolute CD4^+^ and CD8^+^ T cell numbers in the blood at baseline and sacrifice in Figure S2A.

*Fig.S1D, not many mice were EBV-positive, it would be better to represent the viral titers and not just positive vs. negative.*

We now present the raw EBV viral titers in CNS-derived lymphocytes in addition in Figure S1E.

*Where were the tumors located? What was their size? Is other information available about them?*

Tumors were found most frequently in the spleen, pancreas and within the visceral fat of the abdomen in proximity to the pancreas. Of note: it can be hard to distinguish tumors located in the pancreas versus the visceral fat macroscopically at necropsy and depending on the tissue infiltration with EBV^+^ cells and accompanying immune cell infiltrate, histological identification of the specific organ can be challenging. Less frequently tumors were also found in the mesentery or the liver. This information is now conveyed in the main text.

The size of tumors was not measured at necropsy, though no striking differences in sizes were noted between the groups. Biopsies of tumors and spleens were formalin-fixed and paraffin embedded for histological analysis. Based on the transcriptome profiling results, we investigated these sections immunohistochemically and found that tumors harbored both CD30^+^ and EBNA2^+^CD30^+^ cells more frequently compared to non-tumorous spleen tissue (Figure 5D).

*Since only a minority of mice got tumors (Fig. 1E), it would be interesting and important to know whether these were mice that had*

*- higher B cell counts than others, or lower T or NK cell counts at infection?*

We compared baseline total immune cell reconstitution (i.e. before infection) between mice that went on to develop tumors versus those that did not, including all mice for which this data was available and display the analysis in Figure S1I. We did not observe any substantial differences in the quantified human immune cell populations before infection.

*- higher FK506 plasma levels?*

We now display a direct comparison of FK506 levels in mice with and without tumor formation in the supplementary Figure S1F. We did not observe a difference in FK506 levels.

*- higher EBV load in blood or spleen?*

We now display a direct comparison of EBV burden in blood and spleen in mice with and without tumor formation in the supplementary Figures S1G and H, respectively. Indeed, tumor presence coincided with significantly higher viral loads. This is an observation we have also made several times in other experiments with EBV-infected humanized mice.

Additional text relating to the above three points has been included in the manuscript: Tumor-bearing and tumor-free mice had similar FK506 levels (Fig S1F). In general, tumor formation was associated with higher blood and splenic viral burden (Fig S1G-H), but not with differences in the immune cell reconstitution prior to infection (Fig S1I).

*- same or different hHSC source samples than non-tumor mice?*

Tumors were found in multiple experiments performed with different hHSC samples. The mice with tumors were derived from the same hHSC source samples as the non-tumor mice. Based on our experience with huNSG mice (not just with the experiments performed here) we have seen that some hHSC donors or reconstituted cohorts may have a higher or lower tendency to form macroscopically visible tumors. Nevertheless, we observed the same trend in all cohorts: more frequent tumor formation and higher viral loads in the EBV-infected FK506-treated group.

*The answer to these questions could, for example, in part be provided by representing the mice with 0, 1, or >1 tumors by different symbols in Fig. 1B,C,D and Fig S1A,D.*

We now present the tumor status of individual mice with a color code corresponding to the tumor frequency graph in Figure 1E. Clear symbols = no tumor, black = 1 tumor, red = two or more tumors. For comparable depictions across the entire manuscript, we have expanded the Reviewer’s suggestion to Figures 3 and S3 that relate to EBV gene expression.

*Fig. 2A,B,C,D,F, S2B,C,D,G,H, please show the actual cell numbers instead of normalized "fold over mock". That's more informative.*

We do not necessarily agree that the actual cell numbers (non-normalized pooled data) is more informative in this case. When pooling data from different humanized mouse experiments we observe significant cohort (i.e. CD34^+^-donor)-dependent variance in the reconstituted immune parameters. Pooling of raw non-normalized data can obfuscate experimental effects very quickly. Please see Figure R1 at the end of this document for illustration of the following example: We see a difference in the number of HLA-DR^+^ CD8 T cells between EBV-infected and non-infected mice in two depicted independent experiments, conducted with separate humanized mouse cohorts each reconstituted with CD34^+^ cells derived from two different donors. The effect of EBV infection on the number of HLA-DR^+^ CD8 T cells is similar in each individual experiment. However, there is a cohort dependent difference in baseline HLA-DR expression, as such the raw pooled data will incorporate not only the variance associated with the effect of EBV, but also the variance between the cohorts. In the B cell profiling dataset analysis we addressed this problem via application of surrogate variable analysis. For certain immune parameters we think a normalization step (to the non-infected non-treated group) is an appropriate though certainly not perfect solution to address inter-cohort variance in humanized mouse research. It is important to note that the data we present retains the variance derived from the total cell numbers and the magnitude of differences between the groups of individual experiments. It does not represent percentage-based analysis of flow cytometry populations.

We had included raw non-normalized numbers of CD4 and CD8 T cells per ml Blood in Figure S2A, this is easier to do as baseline and sacrifice values can be paired per mouse. In order to try to comply with the Reviewer’s request and provide the reader with additional information we now provide the absolute cell numbers from a representative experiment (a single experiment performed with CD34^+^ cells from a single HPC donor) in the new Figure S2B.

*Fig.2E shows evidence of T cell "attenuation" due to FK506, but only in the Mock situation (not the EBV situation). What is the p value for this effect? It's missing in the diagram.*

We have included the p value as requested. However, as this is an analysis of reactivity toward EBV^+^ LCLs in non-infected mice, we are of the opinion that the observed effect of FK506 treatment should be interpreted with caution.

*- line 138, "a similar effect ... (Fig. S2B)", maybe, but not significant in this case, please amend.*

*-line 140, "whereby more central- or effector memory T cells were found", yes, but only in some of the comparisons, e.g. in Fig. 2B, CD4+, mock-FK506 vs EBV-FK506, there was no difference. The following sentence ("Yet, in...") does not really clarify this.*

*- line 143, "clearly diminished", again, this was seen only for some of the conditions shown, not for CD8+EBV±FK506 in blood and spleen (Fig 2C and S2D) and not for CD4+ in the same condition in spleen (S2D). The following sentence ("However,...") seems to acknowledge this in some way, but is not precise enough (CD4+EBV spleen were not reduced by FK506), and also seems to partially contradict the previous sentence. Overall, the description in the text is not clear enough here.*

We have rewritten this part of the text in an effort to be clearer. It now reads as follows:

A similar but non-significant effect on the CD4^+^ T cell composition was observed in the spleen. To investigate the pattern of expansion in more detail, we characterized the differentiation status of T cells, whereby more central- or effector memory CD8^+^ T cells were found in EBV-infected animals with and without FK506 treatment (Figs 2B and S2D). The slightly lower level of CD4^+^ T cells observed in the blood of EBV-infected FK506-treated animals in comparison to their non-treated EBV-infected counterparts was likely due to a lack of expansion of CD4^+^ T cell memory compartments. FK506 clearly diminished the number of activated HLA-DR^+^ T cells in non-infected mice (Figs 2C and S2E). However, in EBV infected mice, FK506 treatment restrained the expansion of activated CD4^+^ T cells but not activated CD8^+^ T cells in the blood.

*line 147, "steady state conditions" seems to be a synonym for mock infection but is not a very suitable one, since the mock-infected mice were not that steady in their state either (see S1B,C, mortality and weight loss).*

We agree with this comment and have rewritten this part of the text. It now reads: Consistent with an interrupted TCR signaling pathway, the serum levels of IL-2 were decreased in FK506-treated compared to non-treated animals with or without EBV infection.

*line 150-164, the situation is quite complex, and so is the present interpretation. The argument in favor of curbed CD8+ responses is sophisticated but not entirely convincing. Major findings were that CD8+ responses and EBV-specific responses were consistent in being not significantly reduced by FK506 in the EBV situation (2A,C,E, S2B,D). In spite of this, the authors seem to suggest that the "positive correlation between activated or total CD8+ T cells and the rate of IFNγ-secreting T cells in EBV-infected animals but not in those treated with FK506 (Figs 2F and S2H)" is an argument in favour of specific T cell attenuation. However, since in the FK506 situation (2F, right; S2H, right) the sample number was smaller (n=6), it was more likely to result in non-significance. It is always difficult to draw positive conclusions from the absence of significance, especially if the sample size is small.*

We have now performed additional experiments and repeated the correlation with more mice. We now analyzed 9 mice in each group. Once again we see a strong correlation between the rate of IFNγ-secreting cells and the amount of splenic CD8^+^ or HLA-DR^+^ CD8^+^ in the EBV+ situation (spearman r > 0.9, p < 0.001). But in the EBV^+^ FK506^+^ group we could not see a correlation between these parameters. We present this data at the end of this document for the reviewer in Figure R2.

However, we understand that this argument, though suggestive of a disruption of T cell function in some manner, is not a direct argument in favor of attenuated EBV-specific T cell function *in vivo*. As such, we decided to investigate this point further. We agree with the reviewer that the Elispot assay essentially shows similar levels of EBV-reactive cells in both EBV-infected groups (FK506^–^ and FK506^+^) and report this as such in the revised manuscript. We postulated that the *ex vivo* assessment of the T cells via Elispot would likely measure the number of EBV-specific cells but not accurately reflect the *in vivo* functionality in line with previously published data on *ex vivo* T cell proliferation from tacrolimus-treated pediatric kidney transplant recipients (Laskin et al., Tranplant Direct 2017). Since the FK506 compound was likely cleared from the splenocyte suspension due to multiple washing steps during cell purification from the spleen. Indeed once FK506 was supplemented to the T cell / LCL co-culture at a concentration in line with the measurements of drug levels *in vivo* (20ng/ml), the EBV-specific response was significantly lower compared to culture conditions with carrier solution supplementation (based on raw SFC numbers). Upon normalization to the condition without *in vitro* FK506 supplementation, we could observe that the percentage decrease in SFC varied strongly between mice but was similar between groups with and without FK506 treatment *in vivo*. A trend toward a more pronounced percentage decrease in cells derived from mice that had not had *in vivo* treatment may be speculated. We find that the inhibition of responses via supplementation of *in vivo*-levels of FK506 is a more straightforward argument in favor of attenuated CD8^+^ EBV-specific response *in vivo*. This data is now depicted in figure 2E-F with a positive control experiment in S2I and is discussed in the paragraph as follows:

Upon sole EBV infection activated and total CD8^+^ T cell numbers correlated positively with the EBV viral burden in the spleen, whereas in EBV^+^ FK506^+^ mice no such correlations could be observed, indicating that the higher antigenic burden during treatment did not lead to further expansion of activated CD8^+^ T cells (Figs 2D and S2H). In order to estimate the frequency of EBV-specific T cells, we assessed the number of IFNγ-secreting cells upon contact with autologous EBV-transformed B cells (LCLs) in individual mice from the different groups. We found similar rates of LCL-reactive cells in EBV-infected mice with and without FK506 treatment upon *ex vivo* co-culture (Fig 2E). We postulated that the *in vivo* suppressive effect of FK506 on EBV-reactive T cell function was not retained *ex vivo* due to washing out of the compound during cell isolation, as previously reported for human samples [28]. Indeed, upon supplementation of FK506 at a concentration in line with the compound levels measured *in vivo* (20ng/ml), the EBV-specific response was significantly lower compared to culture conditions with carrier solution supplementation (Figs 2F and S2I). As such, despite similar rates of LCL-reactive cells in FK506-treated and non-treated EBV-infected animals measured *ex vivo*, these cells were likely attenuated *in vivo*.

*Fig. 3A, why were two different statistical tests used in this single analysis?*

Only an unpaired t test with Welch's correction was used. The figure legend has been corrected.

*Fig. 3CD (also Fig. 1D), these data span several orders of magnitude and are accordingly represented on a log scale. However, their arithmetic mean is presented. The geometric mean or median would fit the structure of the data better. The arithmetic mean is unduly influenced by isolated high datapoints, as can be seen e.g. in Fig.3D LMP1 EBV. Since the non-parametric Mann-Whitney test was used, the median would probably be the most adequate.*

We agree with the Reviewer and have made the suggested changes. We now depict the data median in Fig. 3CD and in Fig. 1D. We have expanded Reviewers suggestion to include other data depictions of similar structure such as Fig. S3, Fig. 1C and S1G and H.

*line 176, "equalized", this seems to simplify the real situation. In fact, normalization to EBER1 made it even clearer that three values stood out in the EBV+FK506 condition for each of the three transcripts EBNA2, LMP1, LMP2, and maybe even EBNA1 Cp/Wp. It seems likely that these three samples are the same for the different transcripts? If correct, it would be good to let readers know. It would be interesting to know whether the latency III pattern was synchronously induced in some samples but not others, in what looks like a bimodal distribution. Were some of the "latency III high" mice carrying tumors?*

Two of the three samples that were highest upon normalization in Figure S3B also had high overall levels in Figure 3 the other did not. One of these animals had a tumor. We have now colored individual mice in Figure 3 and S3 based on tumor presence, this highlights the higher overall latency III transcript levels in Tumor-bearing mice in Fig. 3D, in line with the higher viral DNA loads observed in Fig. S1G-H. No clear association between tumor presence and *EBER1*-normalized transcript values can be seen in our opinion. There is a large spread in the Fig S3B data set in both groups and we could also not observe a significant difference between the groups overall that would indicate a global shift in latency patterns.

The three EBV-infected FK506-treated mice identified by the reviewer in Figure S3B may have higher latency III transcript levels relative to *EBER1* compared to other mice in the same group, however certain mice in the EBV^+^ FK506^–^ group have similarly high transcript levels. Currently we do not believe that we have sufficient evidence for additional Latency III pattern “induction” in cells of individual mice, though this would be interesting to investigate in future studies. This would, however, have to be performed with single cell analysis and be followed up on with detailed histological analysis but can not be retrieved from the analysis of RNA from bulk B cells.

Based on our previous experience characterizing EBV latency in EBV-infected humanized mice we are of the opinion that by far the most common phenotype of infected cells is an EBNA2^+^ LMP1^+^ or an EBNA2^+^ LMP1^–^ phenotype, the latter likely reflecting freshly infected cells (Figure 4F and Figure S3B in McHugh et al., Cell Host & Microbe 2017). This is supported by the fact that Qp-promotor driven EBNA1 is only rarely detected (and at very low levels) in EBV-infected mice (this manuscript, Figure 3B-C).

As such we still believe that higher transcript levels in the EBV^+^ FK506^+^ group (Figure 3D) is a result of a higher number of infected cells rather than a latency pattern shift. The text in the result section has been modified slightly to the following: When we analyzed the expression of EBV latent transcripts associated with the transforming latency III (*Cp/Wp-EBNA1, EBNA2*) and II/III (L*MP1, LMP2A*), we observed significantly increased expression levels of all of these viral oncogenes in B cells of FK506-treated compared to untreated huNSG-A2 mice whereby the highest levels were noted among tumor-bearing mice (Fig 3D). Elevated transcript levels could be due to a higher frequency of infected cells or increased levels of transcription per infected cell. To investigate this, we normalized the transcript levels to *EBER1*, a small non-coding EBV RNA present in all latency patterns. No general difference in the expression of latency III transcripts was detected between the two groups upon *EBER1* normalization. Furthermore, no differences were apparent between tumor-bearing and tumor-free mice (Fig S3A-B). Hence, the increased presence of latency II/III transcripts is likely a result of enhanced accumulation of EBV-transformed B cells, rather than a shift in latency patterns.

*Fig. 4, since the number of samples that underwent this gene expression analysis was necessarily limited (4,5,6,7 samples for the four conditions), it seems important how the samples were chosen. Were these some of the same mice for which data were shown in Fig. 1-3? Which ones? Did the EBV samples include mice with tumors, and how many? Did the samples include mice that had high and low levels of latency III genes (see above, Fig. ), or high and low levels of EBV replication, or high and low levels of CD8 T cells, or activated T cells etc.? If this information is available, it should be revealed to get a better idea how representative these data are of the treatment groups.*

This following information is now provided in the method section: All mice for which B cell RNA was isolated and RNA of sufficient amount was available after EBV transcript RT-qPCR were eligible for Ampliseq profiling (n=31). The five mice with the lowest levels of *EBER1* were not included in the analysis. This resulted in 5,6,7 and 8 samples for the four respective conditions (total = 26).

We now also provide an additional supplemental figure (Fig. S4) depicting all the requested data for the individual mice included in the transcriptome profiling in comparison to the complete data sets from Figures 1, S2 and 3.

*line 203, "This is likely due...", I don't quite understand this explanation.*

We have modified the text in an effort to make this explanation more clear: Despite the high number of individual DEGs in the EBV^+^ FK506^–^ vs. EBV^–^ FK506^–^ comparison, we did not identify many significantly enriched gene sets (Fig S6A, Table S2). We assume that B cell gene sets were not uniformly up- or down-regulated, since the B cell population from EBV-infected mice contained not only EBV-infected but also many non-infected cells nevertheless responding to the ongoing infection of the host with altered transcription.

*line 362 "four or five weeks post infection", but Fig. 1 displays "Day 35" as scheduled day of sacrifice if mice needn't be euthanized earlier. This seems inconsistent, please clarify.*

Latest sacrifice date was day 35, but mice had to be terminated according to our animal welfare protocol if they displayed symptoms and weight-loss before this scheduled time point. Most animals were euthanized on day 35 while many indeed happened to be euthanized on day 28.

The scheme in Figure 1 has been amended to include the sacrifice time-span and the respective method section has been amended and now reads: Animals were sacrificed five weeks post infection or euthanized earlier if necessitated by the laboratory’s animal welfare protocol due to general health symptoms and weight loss over 15%.

The respective main text now reads: Mice were euthanized five weeks p.i. or earlier if animals displayed symptoms and/or weight loss as specified in the laboratory’s animal welfare protocol.

*line 367 "were excluded from the EBV group", but hopefully not included in the non-EBV groups, therefore excluded altogether?*

This has now been clarified in the method section and a more accurate nomenclature was used for the groups analyzed. The group nomenclature has been changed throughout the manuscript and the method section now reads: EBV-inoculated mice with no EBV DNA load in blood and spleen and no EBV nuclear antigen 2 staining in FFPE splenic sections were considered non-infected and analyzed together with PBS-injected animals in the respective non-infected group designated as “EBV^–^ FK506^–^“ and “EBV^–^ FK506^+^”. This was the case in 5 out of 25 mice injected with EBV and 4 out of 28 injected with EBV and treated with FK506 (two-sided Fisher’s exact test, p = 0.72).

*line 371 on B cell purification and RNA isolation, what was the minimum number of CD19+ B cells per sample that was analyzed?*

RNA was extracted from a minimum of 1 million cells per mouse. This information has now been included in the method section.

*line 386, "library size ... was normalized to 1 million" - i.e. reads? How many actual reads were acquired per sample at a minimum?*

This information has now been included in the method section of the manuscript: Average number of mapped reads for all samples was 10.307×10^6^, minimum and maximum were 3.746×10^6^ and 29.005×10^6^ mapped reads, respectively.

**Figures for reviewers**

**Figure R1. Depiction of rational regarding normalization between experiments before pooling data.**

**(A-B)** Two independent experiments (A and B) performed with humanized mouse cohorts derived from separate donors (X and Y) with different baseline levels of activated CD8 T cells. Each individual experiment shows a similar effect size on activated CD8^+^ T cell number when comparing EBV infection versus non infection and most EBV^+^ mice show a higher number of activated CD8^+^ T cells compared to non-infected animals. **(C)** Data from both experiments was pooled without normalization: only a minority of EBV^+^ mice shows a difference to the non-infected controls. The effect of EBV-infection on the number of activated CD8^+^ T cells is obfuscated by the inter-donor variance. **(D)** Data from experiments A and B was normalized to the mean of the non-infected groups prior to pooling. The effect size is retained and again majority of EBV^+^ mice display higher activated CD8^+^ T cell levels compared to the non-infected group, which is true to the original data of the individual experiments. This illustration was generated from experimental data included in the main manuscript.

**Figure R2. Correlation between EBV specific response and CD8^+^ T cell expansion.**

EBV-specific T cell response was assessed for individual mice by Elispot assays whereby IFNγ spot-forming cells (SFC) were counted upon co-culture of CD19 MACS-depleted splenocytes with autologous LCLs or medium. Correlations between the number of IFNγ SFC and the relative number of splenic CD8^+^ T cells **(A)** or HLA-DR^+^ CD8^+^ T cells **(B)** are depicted for individual FK506-treated (n=9) and non-treated (n=9) EBV-infected mice. r, spearman correlation. Solid lines represent linear regression trend lines and shaded areas indicate 95% CI of each trend line. Composite data from three independent experiments.

**References**

Chijioke O, Marcenaro E, Moretta A, Capaul R, Münz C. The SAP-dependent 2B4 receptor mediates CD8^+^ T cell-based immune control of Epstein Barr virus in human immune system mice. J Infect Dis. 2015; 212(5):803-7.

Laskin BL, Jiao J, Baluarte HJ, Amaral S, Furth SL, Akimova T, et al. The Effects of Tacrolimus on T-Cell Proliferation Are Short-Lived: A Pilot Analysis of Immune Function Testing. Transplant Direct. 2017;3(8):e199.

Strowig T, Gurer C, Ploss A, Liu YF, Arrey F, Sashihara J, et al. Priming of protective T cell responses against virus-induced tumors in mice with human immune system components. J Exp Med. 2009;206(6):1423-34.
