# Supplementary material for: Immunosuppressive FK506 treatment leads to more frequent EBV-associated lymphoproliferative disease in humanized mice
Source: PLoS Pathog. 2020 Apr 6;16(4):e1008477. doi: 10.1371/journal.ppat.1008477 (PMC7162544; doi:10.1371/journal.ppat.1008477)
Supplement: Attachment — Submitted filename: point-by-point 110320.docx [file ppat.1008477.s014.docx]

Point by point response

*Reviewer remarks in Black italics.*

Author’s response in Green

**Revision #2**

**Part I – Summary**

Please use this section to discuss strengths/weaknesses of study, novelty/significance, general execution and scholarship.

**Reviewer #1:** *This paper examines the effect of an immunosuprressive NFAT inhibitor (FK506), used to inhibit T cell function in transplant patients, on the ability of EBV to induce lymphomas in a humanized mouse model. Not surprisingly, inhibiting T cell function results in increased lymphomas. This has previously been shown to be the case in EBV-infected humanized mouse models when T cells are depleted. The authors also show that a known EBV target cellular gene (CD30) is increased in animals with EBV induced tumors. As stated in my previous review, the results are not particularly novel. The revised manuscript does not change this observation.*

**Reviewer #2:** *The authors have developed a preclinical model to study EBV-PTLD in HLA-A2 transgenic humanized mice. They have performed very thorough studies on analyzing immune responses to EBV in these mice. While the current findings cannot be considered novel (soluble CD39), believe that the model will be of great value in the future as more targeted therapies to treat PTLD are being developed. In addition, believe that it will be of high interest to scientist, who study EBV and EBV-specific immune responses.*

**Reviewer #3:** *The authors have very thoroughly and successfully revised their manuscript. I am indebted to the authors for their detailed explanations and additional information. I have no further requests.*

**Reviewer #4:** *This revised manuscript by McHugh et al described the use of the CD34 humanized mouse model to study the effects of EBV infection and FK506 treatment as a model for post-transplant lymphoproliferative disease (PTLD) in humans. The authors have demonstrated that the humanized mice infected with EBV and treated with FK506 have compromised T cell immune responses, leading to higher EBV viral load, and higher risks in developing tumors. The authors further analyzed the transcriptomes of the treated and infected groups with controls to identify the differentially expressed genes, and identify soluble CD30 as a potential marker for tumor development in the humanized mice and PTLD. This revised manuscript have addressed most of the concerns raised by the previous reviews and have added new experiments and analysis of the data. The authors have also re-written parts of the manuscripts to clarify some of the points that were made. The manuscript, as indicated by the previous review, that the study was well designed and conducted, the resulting immunosuppression resulting tumor development in the animals are expected, and represented incremental knowledge gain in the field. The novelty nevertheless, is the use of the humanized mouse model for EBV infected and FK506 treatment, and the identification of sCD30 as potential biomarker for PTLD. There are however, a few remaining points that the authors should address.*

We thank reviewer #4 for taking the time to assess our revised manuscript and agree with the summary provided. We hope that we were able to completely address the remaining points mentioned by the reviewer by this second round of revision experiments. We believe the additional input has strengthend the overall manuscript.

**Part II – Major Issues: Key Experiments Required for Acceptance**

Please use this section to detail the key new experiments or modifications of existing experiments that should be absolutely required to validate study conclusions.

Generally, there should be no more than 3 such required experiments or major modifications for a "Major Revision" recommendation. If more than 3 experiments are necessary to validate the study conclusions, then you are encouraged to recommend "Reject".

Reviewer #1: (No Response)

Reviewer #2: none

Reviewer #3: (No Response)

Reviewer #4: *The demonstration of soluble CD30 as potential biomarker in PTLD patients to confirm the animal model results is one of the most significant findings of the study. However, while the results were statistically significant when comparing the sCD30 levels between diagnosis and recovery, the sample size (n=8) is still small. Several of the 8 samples analyzed have low levels initially and the decrease seems insignificance. Thus, a larger sample size needs to be analyzed. Moreover, more importantly, the study lacks proper control, the levels in normal non-disease individual needs to be determined.*

We have now increased the PTLD sample size from 8 to 13, in each of these cases serum sCD30 levels were measured during the acute phase of PTLD (during or shortly after diagnosis) and again at a recovery timepoint at least 36 months later. Though this cohort of pediatric liver transplant patients can still be considered small, it is valuable due to the longterm follow-up. The new results still indicate that sCD30 may serve well as a biomarker whereby the presence of high levels in the serum will be most informative. It is true that some patients had low levels of sCD30 at diagnosis and as such low levels would clearly not be helpful to rule out PTLD.

In order to provide the requested non-diseased controls we measured sCD30 in 31 healthy children undergoing elective tonsillectomy with similar age and female-to-male ratio as the PTLD patients (detailed in Table S4). Additionally, we measured sCD30 in 21 pediatric patients which presented with non-EBV-associated acute febrile illnesses (again with similar age and female-to-male ratio as the PTLD patients). Half of each of these control cohorts were EBV VCA IgG positive indicating past EBV infection and we saw no difference in sCD30 levels between EBV seropositive and negative individuals. Importantly, acute timepoint PTLD samples had significantly higher levels of sCD30 compared to either of these control cohorts and upon recovery sCD30 returned to levels observed in the healthy control samples.

The results of the sCD30 testing in pediatric PTLD patients and controls is displayed in the revised Figures 5C and S7C and reported in the Results section as follows:

However, investigation of the serum of pediatric liver transplant recipients indicated that PRR4 levels would likely not serve as a useful biomarker for detecting PTLD (Fig S7C). In contrast, levels of sCD30 were significantly higher in PTLD patients at or shortly after diagnosis compared to the same individuals upon recovery (Fig 5C and Table S3). In control cohorts of healthy children undergoing elective tonsillectomy and children that presented with acute non-EBV-associated febrile illnesses the serum concentration of sCD30 was relatively low. Furthermore, sCD30 levels were similar between the EBV VCA-IgG seropositive and seronegative controls (Fig S7D and Table S4).

**Part III – Minor Issues: Editorial and Data Presentation Modifications**

Please use this section for editorial suggestions as well as relatively minor modifications of existing data that would enhance clarity.

Reviewer #1: (No Response)

Reviewer #2: none

Reviewer #3: (No Response)

Reviewer #4: The authors did not fully addressed the former review’s concern on the need to analyze the data for the EBV infected and treated mice that developed tumors versus those that did not, as an additional variable. The authors have provided information in comparing animals with and without tumors in figure 1 but have not compare their differences in immune cell populations and transcriptome profiles. There were a number of animals that were infected and treated with FK506 did not develop tumors but have similar viral loads in spleen and blood as those that developed tumors, the differences in a majority of the infected and treated animals that developed tumors versus those that did not need to be investigated and analyzed further.

We have now compared EBV-infected and FK506-treated animals with and without tumors with regard to their immune cell populations at sacrifice (A) and their B cell transcriptome profiles (B).

A) We have now included the analysis of peripheral blood and splenic CD4^+^ and CD8^+^ T cell counts at the end of the experiment between tumor-bearing and tumor-free mice of the EBV^+^ FK506^+^ group in the new Figures S2F-G. There is a trend to more (HLA-DR^+^) CD8^+^ T cells in the spleen of mice with tumors but not in the blood. This is a finding that we have also observed over many experiments with EBV-infected non-immunosuppressed humanized mice that are not part of this manuscript. We discuss the new analysis in the result section as follows: “Upon sole EBV infection activated and total CD8^+^ T cell numbers correlated positively with the EBV titers in the spleen, whereas in EBV^+^ FK506^+^ mice no such correlations could be observed (Figs 2D and S2J). However, within this group, tumor-bearing mice, which had similar levels of CD4^+^ T cells, had higher numbers of (activated) CD8^+^ T cells in the spleen compared to tumor-free mice (Figs S2F-G).”

We now also display the analysis of the B cell counts at the end of the experiment between tumor-bearing and tumor-free mice of the EBV^+^ FK506^+^ group in the new Figures S3A-B. There was no difference in peripheral blood and splenic B cell counts between the two groups. We have included this information in the result section. It now reads: “EBV-driven PTLD in patients display latency II or III associated gene expression [1]. In order to characterize the viral latency pattern upon FK506 treatment *in vivo*, we analyzed the EBV-specific gene expression in CD19^+^ splenocytes. Firstly, we found that FK506 treatment does not influence the abundance of B cells in the spleen with or without EBV infection (Fig 3A) and B cell counts were similar between tumor-bearing and tumor-free mice of the EBV^+^ FK506^+^ group (Figs S3A-B). Furthermore, FK506 did not affect the frequency of detection or the relative expression of the latency I/II-associated *Qp-EBNA1* or the immediate early lytic transcript *BZLF1*, respectively (Figs 3B-C).”

Reviewer #3 had previously asked if there was a difference in immune cell populations prior to infection between mice that later on developed tumor(s) and those that did not. This question we addressed in Supplementary Figure S1I in the last round of revision.

B) In addition to immune cell counts, we compared the host transcriptome of CD19^+^ splenocytes isolated from tumor-bearing and tumor-free mice within the EBV^+^ FK506^+^ group, as suggested by Reviewer #4. The differential gene expression analysis did not yield any signifinanly up- or down-regulated genes.

Furthermore, as visualized on the PCA plot below (Fig R1), there is no apparent separation of tumor-negative (2) and tumor-positive (6) mice. However, it is important to note that our experiments were not devised to analyse the differences between tumor-bearing and tumor free mice within the same group, but rather to find differences and similaritries between the different experimental groups. We cannot rule out that differences in host gene expression could be found if experimental setup and analysis were structured to address that specific question (i.e. many more mice with and without macroscopicaly visible tumors from this specific group). The information on macroscopic tumor presence has been added to the metadata of the transcriptome datasets available to the reader on the Gene Expression Omnibus (GEO) database (https://www.ncbi.nlm.nih.gov/geo/) with the accession number GSE126515.

DEA: Tumor-positive vs. negative

```
## Down        0
## NotSig      11743
## Up             0
```

**Figure R1. Host transcriptome analysis with stratification according to tumor presence within EBV^+^ FK506^+^ group.** Differential expression analysis (DEA) was performed using mixed-effect linear regression model with empirical Bayes variance estimation (limma package), as described in the methods. Principal component plot: Sample similarity in a 2D projection by multi-dimensional scaling of human transcriptome profiling from splenic B cells isolated from animals in the EBV^+^ FK506^+^ group. Spurious batch effects of unknown origin and HFL donor-specific effects were corrected by surrogate variable analysis.
